# Exploratory Radiomic Analysis of Conventional vs. Quantitative Brain MRI: Toward Automatic Diagnosis of Early Multiple Sclerosis

**DOI:** 10.3389/fnins.2021.679941

**Published:** 2021-08-05

**Authors:** Elizaveta Lavrova, Emilie Lommers, Henry C. Woodruff, Avishek Chatterjee, Pierre Maquet, Eric Salmon, Philippe Lambin, Christophe Phillips

**Affiliations:** ^1^The D-Lab, Department of Precision Medicine, GROW—School for Oncology, Maastricht University, Maastricht, Netherlands; ^2^GIGA Cyclotron Research Centre In Vivo Imaging, University of Liège, Liège, Belgium; ^3^Clinical Neuroimmunology Unit, Neurology Department, CHU Liège, Liège, Belgium; ^4^Department of Radiology and Nuclear Imaging, GROW – School for Oncology and Developmental Biology, Maastricht University Medical Centre, Maastricht, Netherlands; ^5^GIGA In Silico Medicine, University of Liège, Liège, Belgium

**Keywords:** multiple sclerosis, brain MRI, radiomic feature, quantitative MRI, histological MRI

## Abstract

Conventional magnetic resonance imaging (cMRI) is poorly sensitive to pathological changes related to multiple sclerosis (MS) in normal-appearing white matter (NAWM) and gray matter (GM), with the added difficulty of not being very reproducible. Quantitative MRI (qMRI), on the other hand, attempts to represent the physical properties of tissues, making it an ideal candidate for quantitative medical image analysis or radiomics. We therefore hypothesized that qMRI-based radiomic features have added diagnostic value in MS compared to cMRI. This study investigated the ability of cMRI (T1w) and qMRI features extracted from white matter (WM), NAWM, and GM to distinguish between MS patients (MSP) and healthy control subjects (HCS). We developed exploratory radiomic classification models on a dataset comprising 36 MSP and 36 HCS recruited in CHU Liege, Belgium, acquired with cMRI and qMRI. For each image type and region of interest, qMRI radiomic models for MS diagnosis were developed on a training subset and validated on a testing subset. Radiomic models based on cMRI were developed on the entire training dataset and externally validated on open-source datasets with 167 HCS and 10 MSP. Ranked by region of interest, the best diagnostic performance was achieved in the whole WM. Here the model based on magnetization transfer imaging (a type of qMRI) features yielded a median area under the receiver operating characteristic curve (AUC) of 1.00 in the testing sub-cohort. Ranked by image type, the best performance was achieved by the magnetization transfer models, with median AUCs of 0.79 (0.69–0.90, 90% CI) in NAWM and 0.81 (0.71–0.90) in GM. The external validation of the T1w models yielded an AUC of 0.78 (0.47–1.00) in the whole WM, demonstrating a large 95% CI and a low sensitivity of 0.30 (0.10–0.70). This exploratory study indicates that qMRI radiomics could provide efficient diagnostic information using NAWM and GM analysis in MSP. T1w radiomics could be useful for a fast and automated check of conventional MRI for WM abnormalities once acquisition and reconstruction heterogeneities have been overcome. Further prospective validation is needed, involving more data for better interpretation and generalization of the results.

## Introduction

Multiple sclerosis (MS) is an inflammatory disorder of the central nervous system, responsible for focal and diffuse damages, including both demyelination and neurodegeneration, and often leading to physical and mental disability (Lassmann, 2018; Chen et al., 2019). In 2016, there were more than two million prevalent cases globally (Wallin et al., 2019). In Europe, the overall mean cost per patient was more than €50K (adjusted to 2015 purchasing power parity) in a severe disease (Kobelt et al., 2017).

Early diagnosis in MS is challenging because the pathology mechanisms are not yet completely understood, and disease biomarker discovery is still ongoing. The McDonald criteria is currently used for diagnosis (Thompson et al., 2018). It assimilates information about clinical relapses and focal white matter (WM) lesions (plaques) visualized with conventional magnetic resonance imaging (cMRI) and cerebrospinal fluid (CSF) analysis (Trip and Miller, 2005; Kaunzner and Gauthier, 2017; Oh et al., 2018; Thompson et al., 2018). If the patient does not meet the diagnostic criteria, the diagnosis of MS is provisionally not retained. Although cMRI is playing a valuable role in routine clinical practice, it merely captures a very small proportion of MS-related pathological processes (Zivadinov and Leist, 2005; Filippi et al., 2019). It is particularly not sensitive to detect and track diffuse pathological changes occurring both in the normal appearing white matter (NAWM) and gray matter (GM). These changes appear in the early stages of the disease and better correlate with clinical outcomes than only the WM focal lesion load (Griffin et al., 2002; Bonnier et al., 2014; Yoo et al., 2018; Davda et al., 2019; Treaba et al., 2019). Additionally, routine cMRI voxel intensities are expressed in arbitrary units, which vary based on a large number of factors, including the patient being examined, equipment, and protocol being used. This makes MRI analysis strongly dependent on the expertise of the medical specialist and hinders data reproducibility and comparison in follow-up and cross-sectional studies. Therefore, there is an unmet clinical need for the development and automated detection of quantitative and objective early MS biomarkers.

Quantitative MRI (qMRI) potentially overcomes these limitations by quantifying the physical micro-structural properties of brain tissues in standardized units. Commonly, some of the following parameters are estimated: longitudinal and effective transverse relaxation rates (R1 and R2^∗^, respectively) or times (T1 and T2^∗^, respectively), proton density (PD), magnetization transfer (MT) saturation, and a number of diffusion MRI (dMRI) metrics. Values in qMRI maps are linked to the physical properties of biological tissues, such as axonal myelination (MT, R1, R2^∗^, T1, and dMRI), iron accumulation (R2^∗^ and T2^∗^), and free water proportion (PD) (Weiskopf et al., 2013; Weiskopf et al., 2015; Tabelow et al., 2019). It has been shown that qMRI data are fairly reproducible between different scanners and attractive for multi-center studies (Gracien et al., 2020). Current MS research compares the qMRI properties of brain between healthy control subjects (HCS) and MS patients (MSP) (Hagiwara et al., 2017a; Reitz et al., 2017; Andica et al., 2018; Yoo et al., 2018; Lommers et al., 2019; Saccenti et al., 2019). It has been shown that, with specific qMRI sequences, more MS-related damages can be detected compared with cMRI using similar acquisition times (Hagiwara et al., 2017b). Furthermore, it has been shown that qMRI reveals pathological GM alterations (Lommers et al., 2021) and early MS-related GM changes (Gracien et al., 2016).

**FIGURE 1:**
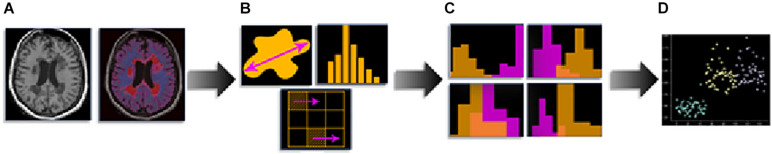
Radiomics pipeline: **(A)** medical imaging and segmentation, **(B)** feature extraction, **(C)** feature selection, and **(D)** modeling.

The discovery of quantitative imaging biomarker is currently experiencing a large increase in research interest, and radiomics is rapidly emerging as a major tool in radiology. Radiomics is a high-throughput imaging data quantification approach aimed to calculate the quantitative descriptors of medical images to characterize the underlying biology and establish a correlation with clinical endpoints (Lambin et al., 2012, 2017a; Rogers et al., 2020). Radiomics has shown promise in personalized medicine for cancer treatment (Coroller et al., 2015; Prasanna et al., 2017; Lambin et al., 2017b; van Timmeren et al., 2017) and is already applied in neurology to predict epilepsy in patients with low-grade gliomas (Liu et al., 2018), to distinguish between MS and neuromyelitis optica spectrum disorders on spine MRI (Liu et al., 2019; Ma et al., 2019), and to differentiate Alzheimer’s disease from mild cognitive impairment on MRI and positron emission tomography (Feng et al., 2018; Li et al., 2019). The standard pipeline for radiomic analysis is presented in Figure 1.

Within the present study, we hypothesized that cMRI- and qMRI-based radiomic models have a diagnostic value in MS, while qMRI-based features have an advantage in the detection of diffuse damages. The objective of the study was to investigate the ability of radiomic features found in WM, NAWM, and GM, extracted from cMRI and qMRI maps, to distinguish between HCS and MSP. Radiomic classification models were developed and tested, and cMRI models were validated on external publicly available datasets.

## Materials and Methods

### Study Design

This study was performed on three datasets: dataset 1 (DS1) contains both cMRI (T1w and FLAIR) and four types of qMRI maps (PD, MT, R1, and R2^∗^) of both MSP and HCS, dataset 2 (DS2) contains cMRI (T1W) of HCS, and dataset 3 (DS3) contains the cMRI of MSP (T1w and FLAIR) (see Table 1). DS2 and DS3 were combined into one validation dataset (DSV) using data selection and additional pre-processing to minimize any mismatch with DS1 regarding demographics and image acquisition parameters. For each participant, the same brain tissue segmentation method was applied. DS1 was randomly split and used to train and test multi-channel qMRI models as well as used for training of cMRI models, while DSV was used to validate the cMRI models. The observations from test subsets were kept apart from those of train subsets and were used only to test the models. For each participant, radiomic features were independently extracted from whole WM, NAWM, and GM regions from all available image types. For MSP, WM volume included combined NAWM and focal WM lesions. Since HCS do not have focal WM lesions, for them WM and NAWM volumes are matching.

**TABLE 1:** Dataset summary details.

	Dataset 1	Dataset 2	Dataset 3
Dataset	Private CHU, Liege	CC-359	MICCAI 2016 MSSEG challenge (training subset)
Participants	MSP (15 relapsing–remitting, 21 progressive), HCS (36)	HCS (359)	MSP (15)
Age, *μ* ± *σ* (years)	45.8 ± 12.1	52.7 ± 7.3	40.5 ± 10.8
Gender, M/F	0.76	0.96	1.00
Image types	T1w, PD, MT, R1, R2*, FLAIR	T1w	T1w, FLAIR
Sites	CHU (Liege, Belgium); GIGA-CRC *in vivo* imaging, University of Liège (Liege, Belgium)	Campinas (Sao Paulo, Brazil); Calgary (Alberta, Canada)	CHU Rennes (Rennes, France); CHU Lyon (Lyon, France)
Equipment	3 T Siemens Magnetom Allegra (37); 3 T Siemens Magnetom Prisma (35)	3 T and 1.5 T Siemens (120), Philips (119), GE Healthcare (120) MRI scanners	3 T Siemens Magnetom Verio (5); 1.5 T Siemens Magnetom Aera (5); 3 T Philips Ingenia (5)
Protocol	MPM protocol with FLASH sequences	3D MP-RAGE (Philips, Siemens), comparable 3D T1w spoiled gradient echo sequence (GE Healthcare)	Sagittal 3D FLAIR, sagittal 3D T1w
Matrix	256 × 224	224 × 224, 240 × 240, 256 × 256	256 × 256 (Siemens), 336 × 336 (Philips)
Slices	176	164–224	176 (Siemens), 200 (Philips)
Voxel resolution (mm^3^)	1 × 1 × 1	1 × 1 × 1 (Siemens)	1.08 × 1.08 × 0.9 (1.5 T Siemens), 1 × 1 × 1 (3 T Siemens), 0.74 × 0.74 × 0.85 (Philips)

**μ, average; σ, standard deviation; M, male; F, female.**

**FIGURE 2:**
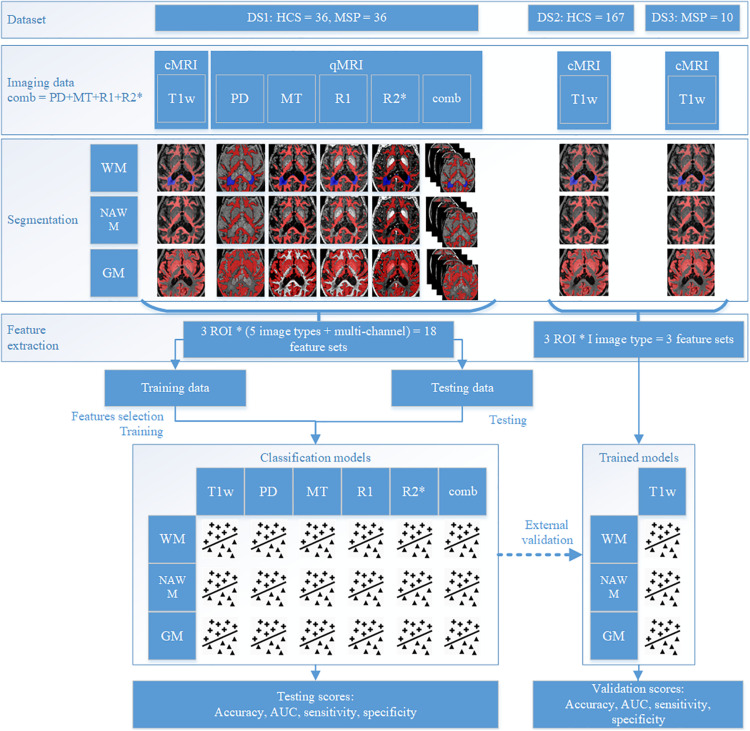
Study design.

With the addition of models combining features extracted from all four qMRI maps, a total of 18 models were trained on DS1 [three regions of interest (ROIs), five image types, and a combination thereof], of which three models (three ROIs, one image type) were validated on DSV. All feature selection and model training were performed in the respective training datasets. The testing and/or validation datasets were kept apart and were used only for evaluation purposes. The study design is detailed in Figure 2. For each step, workflow execution times were recorded, and the averages reported.

### Data Description

**FIGURE 3:**
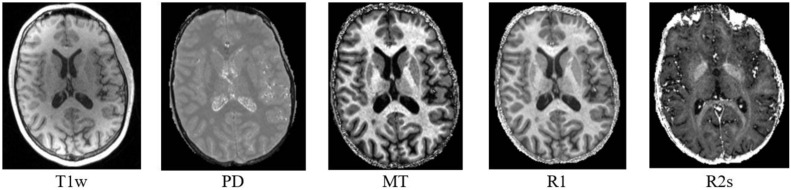
Example of MRI data presented in DS1.

DS1 is a private dataset consisting of 72 participants, 36 MSP with relapsing–remitting and progressive forms (CHU Liege, Belgium), and 36 HCS (GIGA-CRC *in vivo* imaging, University of Liège, Liege, Belgium) acquired within an MS cross-sectional study (local ethic committee approval B707201213806) retrospectively collected between 2013 and 2017 (Lommers et al., 2019). It contains cMRI data (T1w for all the participants and FLAIR only for the MSP) and qMRI maps (PD, MT, R1, and R2^∗^; see Figure 3). The inclusion criteria were as follows: (1) age between 18 and 65 years, (2) Expanded Disability Status Scale (EDSS) not more than 6.5, (3) no relapse in the previous 4 weeks, and (4) MRI compatibility. The details of the MPM protocol are available in Lommers et al. (2019). MS status was estimated by CHU Liege neurology specialists based on McDonald’s criteria 2010 (Polman et al., 2011). This dataset was used for all the exploratory analyses, including feature selection and model parameter tuning. Before the feature selection and subsequent steps, DS1 was randomly split into training and testing subsets (80/20%), attempting to maintain distributions of outcome, age, gender, and scanner variables.

DS2 is the Calgary–Campinas-359 dataset—an open, multi-vendor, multi-field-strength brain MRI dataset (Souza et al., 2018). It is composed of volumetric T1w images of 359 presumed healthy adults, scanned between 2009 and 2016. In the dataset description, there is no information about the neurological status assessment.

DS3 is a subset of the MICCAI 2016 MS lesions segmentation (MSSEG) challenge dataset. The MSSEG challenge dataset contains MRI data for 53 MSP, but only 15 participants from the training subset are publicly available (Cotton et al., 2015; Commowick et al., 2018). The data were acquired not later than 2016 in three different sites in France on four different multi-field multi-vendor scanners with different sequences, including T1w and FLAIR. We used the unprocessed data from DS2 to implement the same image pre-processing protocol for all the datasets.

There are some differences between DS1 and DSV, the main difference being the different image acquisition equipment and protocols (see Table 1). Other differences are the lack of information about how HCS and MSP status, respectively, was assessed in DS2 and DS3, and the lack of MS stage of EDSS in DS3, making a comparison between DS1 and DS3 difficult. To minimize those differences and any potential bias, DS2 and DS3 were combined and filtered to match the age range and field strength present in DS1. Within the datasets, there were no incomplete data.

A summary of the datasets is presented in Table 1.

### MRI Data Pre-processing

All the data processing and analysis hereafter were performed on a system containing 4 × 10 core 2.40 GHz Intel Xeon CPU and 64 GB RAM.

The qMRI maps were generated in MATLAB 2017b (The MathWorks Inc., Natick, MA, United States) with the use of the hMRI toolbox, v0.2.0 (Tabelow et al., 2019), an extension of SPM12^1^. In the absence of radiofrequency field sensitivity bias map acquisition, the radiofrequency field bias was corrected with a unified segmentation approach. The radiofrequency transmit field (*B*_*1*_) bias was corrected using B1 and B0 maps, which were acquired with 3D echo-planar imaging mapping protocols. The B1 data was processed with parameters which were identical to the standard default ones. The multiparameter input images included six MT-, eight PD-, and six T1-weighted images.

All images within DS1 were reconstructed with a resolution of 1 × 1 × 1 mm^3^; hence, we decided to resample the scans within DS2 and DS3 to the same resolution. We used cubic spline interpolation as it performs well in terms of its Fourier properties, visual image quality, and interpolation errors (Lehmann et al., 1999).

Following this step, tissue masks for CSF, GM, NAWM, and lesions within DS1 were estimated. Tissue segmentation in HCS was performed with a multi-channel unified segmentation protocol (Ashburner and Friston, 2005), using multiple qMRI maps (PD, MT, R2^∗^, and R1). It was performed in MATLAB using hMRI for SPM12 with light regularization (regularization coefficient, 0.001) and 60-mm cutoff for full-width at half-maximum of Gaussian smoothness of bias. The outputs were tissue probability maps for CSF, GM, and WM, with the voxel values between 0 (background) and 1 (corresponding brain tissue). In order to ensure the inclusion of only the relevant tissue class, binary masks for each tissue were obtained by thresholding the tissue probability maps at a high level of 0.9. For MSP, lesion masks were generated from the combination of T1w and FLAIR images with LST (Schmidt et al., 2012)^2^ for SPM12 by the lesion growth algorithm and corrected manually by a qualified MS specialist (ELo) when necessary. Multi-channel tissue segmentation was performed using multiple qMRI maps (PD, MT, R2^∗^, and R1) with unified segmentation protocol in US-with-Lesion (Phillips and Pernet, 2017)^3^, adding an extra lesion tissue class. In DSV, brain tissue segmentation was performed with a single channel (T1w) unified segmentation protocol in MATLAB with SPM12, using T1w images.

After segmentation, total intracranial volume (TIV) was estimated for each patient as the morphological sum of the CSF, GM, NAWM, and lesion volumes (where applicable). This combined ROI was used for intensity normalization, as described below.

As the magnetic field inside an MRI scanner is not ideally homogeneous and is affected by objects within it, a bias field signal is introduced, degrading image quality as a smooth, low-frequency signal that distorts segmentation results and feature values. To partially correct for this in T1w images, N4 bias field correction (Tustison et al., 2010) was performed in TIV.

As cMRI voxel intensities are expressed in arbitrary units, the Image Biomarker Standardization Initiative (IBSI) recommends using normalization for raw MR data (Zwanenburg et al., 2016). Therefore, within each T1w scan, the intensities were normalized to arrive at a mean of 0 and a standard deviation of 1. Normalization was performed within the TIV, considering only TIV intensities.

### Radiomic Feature Extraction and Exploration

Radiomic features that quantitatively characterize the ROI, e.g., intensity histogram, simple statistics, and texture (Lambin et al., 2012; Rizzo et al., 2018), were extracted from pre-processed cMRI and qMRI data using PyRadiomics 2.2.0 (van Griethuysen et al., 2017) in python v.3.7.1. Due to their small volumes, features from lesion ROIs were not extracted, and they were used only as an additional tissue class for brain segmentation. The radiomic features of the following classes were extracted from original images: FO statistics, gray-level co-occurrence matrix (GLCM) (Haralick et al., 1973), gray-level run length matrix (Galloway, 1975), gray-level size zone matrix (Thibault et al., 2013), neighboring gray tone difference matrix (Amadasun and King, 1989), and gray-level dependence matrix (GLDM) (Sun and Wee, 1983). The full list of the extracted features can be found in Supplementary Table 1. Contrary to oncological radiomic studies where shape features are usually involved (Lambin et al., 2012, 2017a; Rizzo et al., 2018), here only first-order and texture features were considered. Many neurodegenerative disorders have reported volumetric brain changes, showing disease-specific patterns in brain substructures (Jakimovski et al., 2020), which were not delineated in the present study. Moreover, WM volumetric atrophy changes are mostly explained with the presence of lesions (Marciniewicz et al., 2019), which also influence first-order and texture features. Therefore, to further reduce the ratio of the number of features vs. the number of samples, shape features were excluded. Before gray-level texture matrices were calculated, intensity discretization was performed with a fixed number of bins *N*_bins_50, in line with IBSI recommendations (Zwanenburg et al., 2016). The fixed bin number approach groups voxel intensities before discretization, which additionally harmonizes multi-scanner multi-vendor multi-site data.

No feature harmonization methods, such as ComBat (Johnson et al., 2007), were applied across the different datasets because of the small sample sizes and considerable heterogeneity of scanners and protocols. To speed up feature extraction, the ROI was pre-cropped into a bounding box with 5-voxel-width padding. A separate feature set was calculated for each ROI and image type. An overview of the feature sets is presented in Table 2.

**TABLE 2:** Overview of the independent feature sets per participant.

ROI	Image type	
WM (for MSP, NAWM + focal WM lesions)	cMRI	T1w
NAWM	qMRI	PD
GM		MT
		R1
		R2*
In total, three ROIs	In total, five image types	

Feature analysis was performed in the whole DS1 to describe the data; its results were not included into model building. Statistical tests were performed to gauge diagnostic efficacy in such a small dataset. A univariate Mann–Whitney test was carried out using Bonferroni correction, and *p* ≤ 0.01 for two-sided hypothesis was considered statistically significant. Point-biserial correlation coefficients *r*_*pb*_ and *p*-values were calculated between radiomic feature values and MS status; a correlation was considered statistically significant if |*r*_pb_| ≥ 0.85 and *p* ≤ 0.05. Spearman correlations between the features and age and the feature ROI volume were computed to gauge the added value of radiomic features compared to age and volumetry, with |*r*_S_| > 0.85 considered highly correlated for each test. Additionally, the univariate area under the receiver operating characteristic curve (AUC) was calculated for each feature.

### Radiomic Feature Selection

In order to remove redundant and non-informative features, feature reduction and selection were performed on DS1, using the MS status as the binary outcome where applicable. Feature selection was independently carried out for the T1w, PD, MT, R1, and R2^∗^ maps to arrive at a subset of *N* features each, attempting to adhere to published rules of thumb to estimate the optimal number (Hua et al., 2005; Abu-Mostafa et al., 2012). We chose the following approach to estimate the number of features $N_{\text{features}}=int\frac{N_{S}}{10}$, as outlined in Abu-Mostafa et al. (2012), where *N*_*S*_ is the number of samples in the minor class.

Since DS1 is relatively small, especially after the train/test split, feature selection as described below was performed 100 times on an extended and balanced cohort of 100 participants created by randomly sampling (with replacement) observations from the training subset. In each of the 100 iterations, a fixed number *N* of the highest-ranking features was retained, and at the end the features were ranked according to how often they were selected.

The feature selection pipeline starts with excluding features with zero or low variance. A feature was considered of low variance if the percentage of its distinct values out of the number of observations was less than 10% and the ratio of its most frequent values was more than 95/5. Next, features with high inter-correlation were excluded by calculating the pairwise Spearman correlation between all the features. From each pair of highly correlated features (| *r*_*S*_| > 0.85), we excluded a feature having the highest correlation on average with all the remaining features. The final selection was performed with recursive feature elimination (Guyon et al., 2002) using random forest classifier (Breiman, 2001) models [100 trees, as recommended by Oshiro et al. (2012); a number of features to consider when looking for the best split $int\left(\sqrt{N_{\text{features}}}\right)$, where $\sqrt{N_{\text{features}}}$ is changing during recursive feature elimination iterations, as recommended by Hastie et al. (2009)]. Random forest (RF) classifiers allow for robust variable importance computation and do not need normalization. Moreover, the number of available features exceeds the number of samples, and a random forest classifier is still able to deal with such data. For each selected feature, a distribution map was generated by calculating the feature value within each 26 connected neighborhood of each voxel within the image ROIs.

### Model Training and Testing

Models were trained and tested on independent subsets of DS1. Observations from the training and testing subsets were randomly sampled with replacement for 100 times, resulting in the creation of extended and balanced training and testing cohorts. Every cohort contained 100 participants.

Separate binary classification models were trained on DS1 for different image types—T1w, PD, MT, R1, and R2^∗^—and for a combination of features from PD + MT + R1 + R2^∗^ (composed of qMRI) to investigate the value of each image type and ROI in the estimation of the MS status. For each image type, three binary classification models were trained using the same features from each image type and ROI: (i) random forest (RF), (ii) support vector machine (SVM) (Platt, 1999), and (iii) logistic regression (LR). For the RF model, the same settings as for the recursive feature elimination were used; for SVM, a radial basis function kernel was used with regularization parameter C = 1.0, kernel coefficient γ = 1/(*N*_features_ ⋅ Var(*X*)), where Var(*X*) is the variance of the input feature *X* (since we did not have any *a priori* expert knowledge about the classification problem and did not perform any empirical validation of the model parameters, these are the default parameters for the SVM, keeping a balance between classification accuracy and tolerance to misclassification errors), and for LR, L2 penalty was used since this regularization does not lead to high values among the regression coefficients, with dual formulation, as recommended when the amount of observations exceeds the amount of features, and a liblinear solver, which is recommended for small datasets; inverse of regularization strength C = 1.0, which is optimal in terms of balance between accuracy and model complexity. Due to the small dataset sizes, DS1 was used again as an exploratory dataset.

The performances of the models were estimated in terms of the following metrics: accuracy, sensitivity, specificity, and AUC, with the corresponding 90% confidence intervals (CI); for each model, learning and curves were plotted. Since all the scores were estimated on the data subsets, containing equal numbers of HCS and MSP, the imbalanced data correction was not needed. The best model was selected based on these performance metrics for different ROIs and tissue types, giving the AUC score more weight and excluding models with median AUC scores below the threshold of 0.7, which is considered an underperforming classification model. In order to select the best model type (RF, SVM, or LR), the number of highest AUC scores was used.

The final models with the original coefficients were subsequently validated on DS2 and DS3. As the combined dataset containing DS2 and DS3 was highly unbalanced regarding the outcome, bootstrapping with balanced sampling was implemented. The models for qMRI were not validated externally due to the unavailability of similar datasets.

To examine the models and methodology for overfitting, a permutation test was performed on DS1. The class labels in both training and testing subsets were randomized, maintaining the same distributions as in the original subsets. Without modifying the pipeline, feature selection was performed, models were trained and tested, and performance metrics were calculated to ascertain whether the pipeline detects patterns in randomly generated outcomes.

## Results

### Data Description and MRI Data Pre-processing

Participants were drawn from DS2, aiming to match DS1 regarding age and magnetic field strength. Participants with MRI quality, which was not sufficient for robust automatic segmentation, were excluded after a visual check (ELa). Finally, 167 participants were selected from this dataset. Another 10 participants were selected from DS3, again trying to match the age and field strength distributions with those of DS1. An overview of the resulting feature sets is presented in Table 3. The *p*-values for comparison of age and gender distributions between HCS and MSP groups within development and validation data as well as between development and validation datasets can be found in Supplementary Table 2. Details of the distribution of participants between the train and test subsets of DS1 and the significance results for comparison of age and gender distributions in the train and test subsets can be found in Supplementary Table 3.

**TABLE 3:** Dataset summary details for the included participants.

	Dataset 1	Dataset 2	Dataset 3
Participants	MSP (15 relapsing–remitting, 21 progressive), HCS (36)	HCS (167)	MSP (10)
Equipment	3 T Siemens Magnetom Allegra (37); 3 T Siemens Magnetom Prisma (35)	3 T Siemens (53), Philips (54), GE Healthcare (60) MRI scanners	3 T Siemens Magnetom Verio (5); 3 T Philips Ingenia (5)
Age, *μ* ± *σ* (years)	45.8 ± 12.1	52.7 ± 7.3	40.5 ± 10.8
Gender, M/F	0.76	0.96	1.00

**μ, average; σ, standard deviation; M, male; F, female.**

### Radiomic Feature Extraction and Description

For each T1w and qMRI image and ROI combination, 93 features were extracted, resulting in 1,395 features per participant. The Mann–Whitney test revealed that 16% of the features (220 features out of 1,395) were sampled from significantly different distributions in the HCS and MSP cohorts, mostly originating from WM in all image types but also from NAWM in MT and R2^∗^. In the entire feature set, there was only one feature (R1 first-order minimum in WM) that was highly correlated with the outcome, no feature was highly correlated with age, and 10 features out of 1,395 were highly correlated with ROI volume. A univariate analysis showed that 28% of the features (395 features out of 1,395) had an area under the receiver operating characteristic curve (ROC AUC) score > 0.75, most of which were obtained from the PD, MT, and R2^∗^ maps (see Table 4).

**TABLE 4:** Number of features out of 1,395 with age, volume, and outcome correlations having an | rS| > 0.85 as well as univariate AUC > 0.75 and corrected Mann–Whitney *p* < 0.01 (listed in Supplementary Table 2).

	ROI	T1w	PD	MT	R1	R2*
$\left|r_{S}^{\text{age}}\right|>0.85$	WM	0	0	0	0	0
	NAWM	0	0	0	0	0
	GM	0	0	0	0	0
$\left|r_{S}^{\text{volume}}\right|>0.85$	WM	0	3	1	1	0
	NAWM	0	3	1	1	0
	GM	0	0	0	0	0
$\left|r_{pb}^{\text{outcome}}\right|>0.85$	WM	0	0	0	1	0
	NAWM	0	0	0	0	0
	GM	0	0	0	0	0
AUC_univar_ > 0.75	WM	13	62	21	45	52
	NAWM	8	28	57	9	37
	GM	3	7	26	5	22
$p_{\mathrm{Mann}-\mathrm{Whitney}}^{\text{Bonferroni}}<0.01$	WM	9	41	10	37	7
	NAWM	0	12	42	5	26
	GM	1	0	18	2	10

### Radiomic Feature Selection

In the training subset of DS1, on average among all the image types and ROIs, 7% from the initial feature set were excluded by the low variance step, followed by 79% exclusion by the high correlation step. The number of features per set kept after each feature selection step is available in Supplementary Table 4. The RF-based recursive feature elimination using data sampling with replacement yielded the final feature vectors for each ROI and MRI image type. To make the models easier to compare across ROI and MRI image types, the three ($N_{\text{features}}=\frac{N_{\text{S}}}{10}=\frac{28}{10 3}$) top-ranking features were left in each final feature vector. The list of the selected features can be found in Supplementary Table 5. The feature distribution maps are presented in Supplementary Table 6.

**FIGURE 4:**
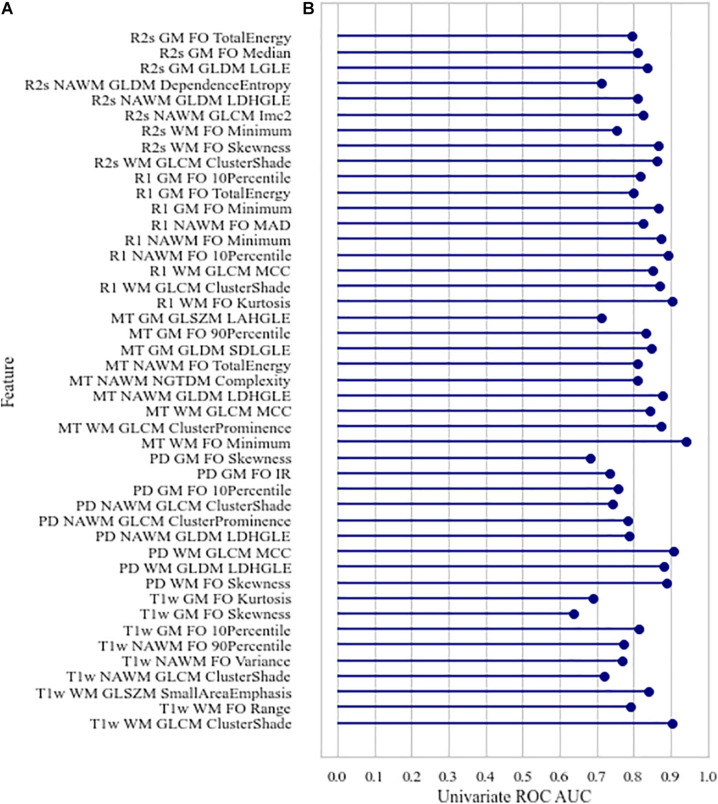
Characteristics of the selected features: **(A)** Spearman age correlation coefficient (absolute) | *r*_*age*_| and Spearman volume correlation coefficient (absolute) | *r*_*volume*_|, **(B)** univariate area under the receiver operating characteristic curve, corrected *p*-values of Mann–Whitney test for differences in value distribution in classes. FO, first order; LDHGLE, large dependence high gray level emphasis; SDLGLE, small dependence low gray level emphasis; LAHGLE, large area high gray level emphasis; MAD, mean absolute deviation; LGLE, low gray level emphasis.

**FIGURE 5:**
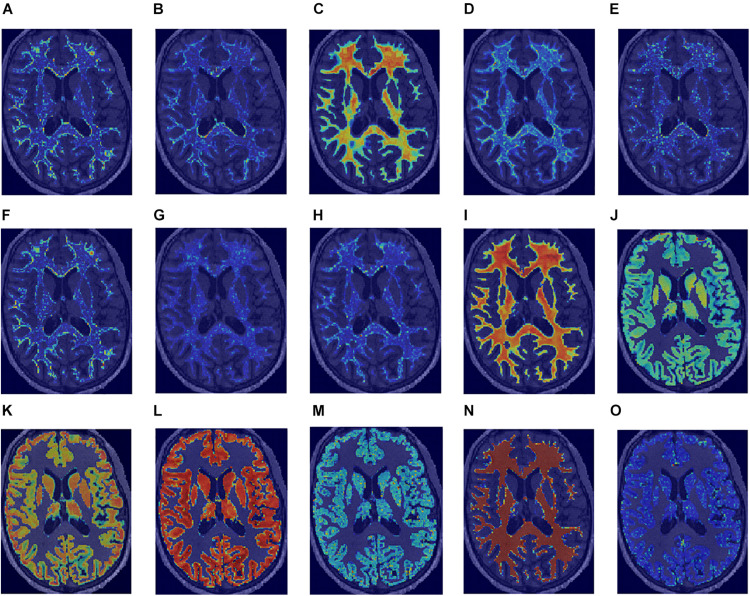
Normalized saliency maps for the best selected features for each region of interest and image type highlight the areas with the highest feature values: **(A)** T1w GLCM cluster shade in WM, **(B)** PD first-order skewness in WM, **(C)** MT first-order minimum in WM, **(D)** R1 first-order kurtosis in WM, **(E)** R2* GLCM cluster shade in WM, **(F)** T1w GLCM cluster shade in NAWM, **(G)** PD GLDM large dependence high gray level emphasis in NAWM, **(H)** MT GLDM large dependence high gray level emphasis in NAWM, **(I)** R1 first-order 10-percentile in NAWM, **(J)** R2* GLCM Imc2 in NAWM, **(K)** T1w first-order 10-percentile in GM, **(L)** PD first-order 10-percentile in GM, **(M)** MT GLDM small dependence low gray level emphasis in GM, **(N)** R1 first-order minimum in GM, and **(O)** R2* GLDM low gray level emphasis in GM.

No high correlations were discovered between the selected features, age, and ROI volume. For the selected features, the univariate AUC was below a threshold of 0.7 for PD, MT, and R2^∗^ in NAWM and T1w and for PD in GM. According to the Mann–Whitney test, the highest number of features with significant differences in means in HCS and MSP is discovered in WM (15 features out of 15) when ranking by ROIs and on R1 (eight features out of nine) when ranking by image types. A list of the selected features with their Spearman correlations with age and the ROI volume, univariate ROC AUC scores, and Mann–Whitney test *p*-value is presented in Figure 4. For the best features in each ROI and image type, saliency maps were obtained by calculation of the feature value in the neighborhood of each voxel. Examples of the normalized saliency maps are presented in Figure 5.

### Model Training and Testing

According to the Delong test with use of the Bonferroni correction, different ML models had significantly different (*p* = 0.01) AUC scores in all the cases, with the exception of MT and qMRIcomb in WM, R1 in NAWM, and PD in GM (the *p*-values for AUC comparison can be found in Supplementary Table 7 and the performance metrics in Supplementary Table 8). Among all the ROI and image types, in most cases, the median values of the RF classifier performance scores dropped below a threshold of 0.7. Having the highest number of top AUC values, the LR model was selected. Results from the LR model will be shown in the main body of the text, while ROC curves and the regression coefficients for the final models are correspondingly shown in Supplementary Figure 1 and Supplementary Table 11. The performance metrics are presented in Table 5.

**TABLE 5:** Logistic regression model performances on testing data showing the median (90% CI) for each image and tissue type (ROI) (median values above 0.7 for all the performance metrics for the same model are highlighted with bold font).

ROI	Image	Accuracy	AUC	Sensitivity	Specificity
WM	T1w	**0.74** (0.66, 0.82)	**0.90** (0.84, 0.95)	**0.76** (0.67, 0.86)	**0.72** (0.59, 0.82)
	PD	0.64 (0.58, 0.71)	0.98 (0.95, 1.00)	1.00 (1.00, 1.00)	0.28 (0.17, 0.42)
	MT	**1.00** (1.00, 1.00)	**1.00** (1.00, 1.00)	**1.00** (1.00, 1.00)	**1.00** (1.00, 1.00)
	R1	0.82 (0.76, 0.88)	1.00 (1.00, 1.00)	0.64 (0.52, 0.75)	1.00 (1.00, 1.00)
	R2*	**0.73** (0.63, 0.83)	**0.86** (0.78, 0.93)	**0.76** (0.62, 0.86)	**0.72** (0.58, 0.84)
	qMRI_*comb*_	**0.93** (0.88, 0.97)	**1.00** (1.00, 1.00)	**1.00** (1.00, 1.00)	**0.86** (0.77, 0.94)
NAWM	T1w	**0.73** (0.66, 0.82)	**0.86** (0.77, 0.93)	**0.76** (0.64, 0.87)	**0.70** (0.59, 0.81)
	PD	0.37 (0.30, 0.44)	0.67 (0.55, 0.81)	0.74 (0.60, 0.87)	0.00 (0.00, 0.00)
	MT	**0.81** (0.74, 0.89)	**0.79** (0.69, 0.90)	**0.76** (0.64, 0.87)	**0.86** (0.77, 0.94)
	R1	**0.87** (0.80, 0.93)	**0.97** (0.93, 0.99)	**0.88** (0.77, 0.98)	**0.86** (0.77, 0.94)
	R2*	0.66 (0.56, 0.76)	0.83 (0.73, 0.94)	0.76 (0.64, 0.87)	0.56 (0.40, 0.72)
	qMRI_*comb*_	0.74 (0.67, 0.81)	0.82 (0.73, 0.90)	0.62 (0.48, 0.77)	0.86 (0.77, 0.94)
GM	T1w	0.41 (0.32, 0.52)	0.60 (0.47, 0.73)	0.26 (0.16, 0.40)	0.56 (0.43, 0.71)
	PD	0.69 (0.61, 0.79)	0.83 (0.74, 0.91)	0.51 (0.38, 0.66)	0.86 (0.77, 0.94)
	MT	**0.88** (0.82, 0.94)	**0.81** (0.71, 0.90)	**0.76** (0.64, 0.87)	**1.00** (1.00, 1.00)
	R1	0.82 (0.75, 0.87)	0.81 (0.72, 0.88)	0.64 (0.50, 0.74)	1.00 (1.00, 1.00)
	R2*	**0.73** (0.65, 0.83)	**0.86** (0.78, 0.95)	**0.76** (0.64, 0.87)	**0.71** (0.58, 0.84)
	qMRI_*comb*_	**0.81** (0.73, 0.88)	**0.86** (0.78, 0.93)	**0.76** (0.64, 0.87)	**0.84** (0.77, 0.95)

Models using features extracted from WM achieved the best classification performance, with the best performance achieved by the MT data. There were no statistical differences (*p* ≤ 0.01) in AUC scores obtained for WM in MT, R1, and qMRIcomb (the *p*-values for AUC comparison can be found in Supplementary Table 9). The highest median performance across all metrics was achieved with the MT model, all of which yielded a value of 1.00. The T1w model performed generally lower than the MT and combined qMRI models but outperformed the PD model in median specificity, the R1 model in median sensitivity, and the R2^∗^ model in median accuracy and AUC.

In NAWM, there were no significant differences in AUC scores obtained for highest scoring R2^∗^ and qMRIcomb models. The highest overall performance was achieved with the R1 model. The PD model yielded a median specificity of 0.00 (no true negatives were achieved). The T1w model performed generally poorer than the MT and R1 models but better than the PD, R2^∗^, and qMRIcomb models.

In GM, there were no significant differences in AUC scores obtained for MT and R1 and for R2^∗^ and qMRIcomb. The highest overall performance was achieved with the MT-based model, which yielded a median accuracy of 0.88.

The permutation test results showed a significant (*p* ≤ 0.01) drop in AUC for all the models, except for PD and MT in NAWM and for T1w in GM. The full results obtained with the permutation test for different models and permutation test *p*-values can be correspondingly found in Supplementary Tables 10, 11.

The classification performance metrics T1w models using the WM, NAWM, and GM validated on the external DSV are presented in Table 6. Since DS2 comprises only MS negative outcomes and DS3 only MS positive outcomes, the separate accuracies for DS2 and DS3 are equal to specificity and sensitivity, respectively, on the whole validation data. Therefore, the medial validation model accuracy for DS2 is 1.00 in WM and NAWM and 0.00 in GM; the medial validation model accuracy for DS2 is 0.30 in WM, 0.20 in NAWM, and 0.90 in GM.

**TABLE 6:** Logistic regression model performances on external validation dataset showing the median (90% CI) for each tissue type for T1w images.

ROI	Accuracy	AUC	Sensitivity	Specificity
WM	0.65 (0.55, 0.85)	0.78 (0.47, 1.00)	0.30 (0.10, 0.70)	1.00 (0.90, 1.00)
NAWM	0.60 (0.55, 0.95)	0.65 (0.29, 1.00)	0.20 (0.10, 1.00)	1.00 (0.90, 1.00)
GM	0.45 (0.15, 0.45)	0.24 (0.05, 0.56)	0.90 (0.10, 0.90)	0.00 (0.00, 0.30)

### TRIPOD Statement and Radiomics Quality Assurance

This study was evaluated with the Radiomics Quality Score (RQS) (Lambin et al., 2017a), which yielded a final result of 39%. We likewise evaluated it with the Transparent Reporting of a Multivariable Prediction Model for Individual Prognosis or Diagnosis (TRIPOD) (Collins et al., 2015) checklist score, which was in the range of 0.71–0.77%. The RQS and TRIPOD checklists are presented in Supplementary Tables 12, 13.

## Discussion

In this exploratory brain tissue MRI and qMRI radiomics study based on a unique dataset, we report on several hypothesis-generating findings for HCS vs. MSP classification. Previous studies on radiomics in MS have been focused on T2w cMRI data and aimed to distinguish between MS and neuromyelitis optica spectrum disorder (Liu et al., 2019; Ma et al., 2019) without external validation, hence the importance of this work.

Of the three machine learning models (RFC, SVM, and LR) tested, LR was the most stable with median accuracy, AUC, sensitivity, and specificity all exceeding a value of 0.7 while achieving the highest performance in terms of AUC. The fact that LR outperformed the other models could be due to the small number of observations, where the simplest models might perform best since they are less likely to overfit. The selected radiomics features were not correlated with age and volume (also a radiomic feature), which indicates that radiomics could provide additional information to those simple variables.

The best LR model performance concerning tissue type was achieved using features extracted from WM. This was expected since focal WM lesions (plaques) in the WM of MSPs affect the intensity distribution (Trip and Miller, 2005). In NAWM classification, which is more challenging, good classification is achieved not only with MT and R1 maps but also with T1w data. This result was not expected since this MRI sequence is not sensitive to pathological NAWM changes within, as reported in Trip and Miller (2005) and Reitz et al. (2017). Nevertheless, it could be explained by the fact that qMRI voxel values have a physical meaning, reflecting the water and myelin contents. Furthermore, the qMRI map generation pipeline contains image co-registration and B0 and B1 fields correction steps, leading to interpolation and, therefore, smoothing of the qMRI map. Consequently, T1w images have a higher spatial resolution, leading to a more detailed texture analysis. In GM, the T1w-based model underperforms, as it was expected, according to previous publications (Trip and Miller, 2005; Reitz et al., 2017).

Among the image types, the best performance was achieved with MT maps, which corroborates the findings of Lommers et al. (2019), where statistical tests showed considerable differences between HCS and MSP. In WM, the MT model demonstrated median accuracy, AUC, sensitivity, and specificity of 1.00, which means that all the training observations were classified correctly. As far as training observations did not enter model training, we can conclude that, in our relatively small dataset, the presence of focal WM lesions (plaques) makes the selected MT features distinctive from the ones extracted from the healthy brain. The PD maps showed the poorest performance with at least one of the performance metrics crossing below a value of 0.7 in each tissue type. This could be due to the potential residual T2^∗^ weighting, as mentioned previously (Lommers et al., 2019). The results obtained with T1w and R1 data were significantly different, although both these image types represent longitudinal relaxation. The main difference between them is that T1w demonstrates the relative level of longitudinal relaxation at some moment, expressed in arbitrary units, whereas the R1 map represents the actual physical property of the tissue and is expressed in standardized physical units (Hz). Furthermore, unlike for T1w data, reconstruction of the qMRI images is always performed with the correction of instrumental biases and receive fields (Tabelow et al., 2019).

Although the T1w models are non-quantitative, they outperformed some of the qMRI models in WM and NAWM yet had the poorest performance in GM. Among all the T1w models, the WM model yielded the highest median AUC of 0.78 and an underperforming sensitivity with a median value of 0.30 on the testing subset of the development dataset. On the external validation for T1w-based models, all showed a poor performance. Nevertheless, among these models, the best performance was achieved in WM, mainly due to the presence of focal WM lesions, which are easily captured in the radiomic analysis. In NAWM and GM, the differences between HCS and MSP are presented on the microstructural level. The T1w data is expressed in arbitrary units, and it is not consistent enough to detect these changes within different scanners and centers. As the T1w-based model in GM underperformed on the testing data, a good performance on the validation dataset was not expected. Thus, even though T1w data can perform well on the development dataset, its application is challenging for multi-centric studies. The explanation can be due to differences in imaging data, lack of sensitivity of T1w contrast for these applications, low predictive ability of the corresponding features, and their susceptibility to data effects. Additionally, we suspect a bias that can be introduced by the clinical differences in the cohorts in DS1, DS2, and DS3. Whereas MS status assessment details, EDSS, and MS stage are known for DS1, there is no such information about the participants from DS3, and there is no information about the tests carried out for DS2 participants to determine them as HCS.

The strengths of the current study include the use of unique quantitative and reproducible imaging data, the use of an external validation open-source data, and in-depth investigation of the features in traditionally challenging tissues such as NAWM and GM, which can have potential in early MS diagnosis.

This study has some limitations, too. The first stems from the small number of observations in the DS1. Consequently, for external validation, we excluded participants which did not correspond to the participants from DS1 in terms of age or MRI magnetic field strength. All participants with insufficient MRI data quality, rendering it unsuitable for robust automatic brain tissue segmentation, were also excluded, introducing more bias. Another limitation is related to the uniqueness of qMRI data, which means that there are no available similar qMRI brain datasets for external validation, especially for MSP. However, it was reported that qMRI is reproducible between different scanner models, and multi-center studies can be expected (Gracien et al., 2020). The third limitation is the absence of data harmonization performed across datasets involved in this study. It results in non-uniformity of non-quantitative MRI data between datasets and thus leads to model performance degradation. The next limitation is related to the analysis of only HCS and MSP data. Although the exploratory analysis of the features demonstrated that some had very high univariate AUC scores (>0.99), absence of data for other neurodegenerative diseases, and relatively small amounts of observations, we cannot conclude that these features themselves can be reliable biomarkers in MS. Thus, an analysis of other neurodegenerative disorders is needed to distinguish between different diagnoses. The fifth limitation pertains to the cMRI sequence analyzed in this study: even though focal WM lesions are noticeable on T1w, this image type is not the leading one in MS investigation. Among cMRI modalities, T2w, FLAIR, and contrast-enhanced T1w provide appropriate contrast. These modalities were not available for all the participants of DS1 (with qMRI acquisition): FLAIR scans were available for MSP only. Therefore, an analysis of another cMRI and qMRI could be a subject of future research. Finally, different brain segmentation approaches were used for DS1 and external validation data. Even though the same method was implemented for all the MRIs, segmentation in DS1 was performed with qMRI data, while segmentation for external validation was performed with cMRI data. It could affect the values of radiomic features, as cMRI-based segmentation leads to an inaccurate delineation of deep GM regions (Nikolaus Weiskopf et al., 2013; Lommers et al., 2019).

Within the present study, we used standard open-source tools for data pre-processing and analysis. Thus, the diagnostic support workflow execution times obtained within this study are indicative. Moreover, they strongly depend on the hardware and software used, original medical image parameters, pre-processing and analysis settings, and radiomic features, composing the final signature. We did not implement any optimization of computational resource consumption; therefore, the obtained execution times represent the upper bound of a workflow duration. Within the present study, cMRI- and qMRI-based workflows took approximately up to 26 and 38 min per participant, excluding the image acquisition time. This difference is due to the relatively long time of qMRI map reconstruction. This shows that the cMRI workflow can be implemented into the brain scanning protocols as a screening for WM abnormalities. The qMRI workflow requires a particular scanning protocol (Weiskopf et al., 2013) and a relatively long analysis time. Therefore, it can be implemented for diagnostic support for patients with suspicious medical evidence.

This study indicated the potential of cMRI and qMRI radiomics in MS-related biomarker development. In differentiating between MSP and HCS, qMRI showed the advantage over cMRI in NAWM and GM regions. Therefore, application of qMRI is promising in early MS diagnosis. We believe that qMRI radiomic signatures can contribute to multi-center studies, as also indicated in previous works (Weiskopf et al., 2013; Weiskopf et al., 2015; Lommers et al., 2019; Tabelow et al., 2019). For this, the reproducibility of qMRI features is to be investigated in the future. T1w WM analysis could potentially be applied for a rapid check of cMRI for WM abnormalities. For research purposes, 7 T MRI is often applied to study NAWM and GM (Treaba et al., 2019; Zurawski et al., 2020), but it is not widely used in clinical practice yet. We believe that 7 T MRI radiomic analysis is a potential research field in MS diagnosis.

Our next step is to validate those findings in a prospective qMRI study and test the hypothesis that those signatures are sensitive to neurodegenerative changes in the early stages of MS and have a diagnostic value for subjects at risk (e.g., clinically isolated syndrome).

## Conclusion

This study demonstrates that brain cMRI and qMRI radiomic features have the potential to distinguish between MSP and HCS. In NAWM and GM analysis, having a potential in early automated diagnosis, stable results are achieved with qMRI-based data. This is a proof-of-concept clinical study demonstrating a strong signal in brain imaging, but further research is needed to develop and approve radiomic signatures for MS.

Nevertheless, future large-scale studies should evaluate the reproducibility and generalizability of the proposed method and create an MS-specific radiomic signature. Because of fully automated pipeline and imaging data quantification, the proposed approach shows its potential in relevance to time-saving and reproducibility in MS diagnosis.

## Data Availability Statement

The data analyzed in this study is subject to the following licenses/restrictions DS2 and DS3 are public datasets, the accession details can be found in Souza et al. (2018) and Li et al. (2019). DS1 MRI data cannot be shared publicly. The code to perform the analysis and radiomic features values are publically available from GitHub URL: https://github.com/CyclotronResearchCentre/brain-tissue-radiomics-on-clinical-and-quantitative-MRI-for-MS (str 1295-1296). The details on the packages, with the indication of versions and functions used, can be found in Supplementary Table 18. Requests to work with the DS1 on a collaborative basis should be directed to ELo, elommers@chuliege.be.

## Ethics Statement

The studies involving human participants were reviewed and approved by the Liege University Hospital-Faculty Ethics Committee, University Hospital Center of Liege, Liege, Belgium. The patients/participants provided their written informed consent to participate in this study.

## Author Contributions

ELa, HW, AC, CP, ES, and PL contributed to study conceptualization and developed the methodology. PL and ES acquired the funding. ELo, PM, and CP performed data acquisition and curation. ELa, HW, and CP performed the analysis and wrote the original draft. HW, CP, PL, and ES performed the supervision. All authors contributed to manuscript revision and have read and approved the submitted version.

## Conflict of Interest

PL reports, within and outside the submitted work, grants/sponsored research agreements from Radiomics SA, ptTheragnostic/DNAmito, and Health Innovation Ventures. He received an advisor/presenter fee and/or reimbursement of travel costs/consultancy fee and/or in kind manpower contribution from Radiomics SA, BHV, Merck, Varian, Elekta, ptTheragnostic, BMS, and Convert pharmaceuticals. PL has minority shares in the company Radiomics SA, Convert pharmaceuticals, Comunicare Solutions, and LivingMed Biotech. He is a co-inventor of two issued patents with royalties on radiomics (PCT/NL2014/050248 and PCT/NL2014/050728) licensed to Radiomics SA and one issued patent on mtDNA (PCT/EP2014/059089) licensed to ptTheragnostic/DNAmito, one non-issued patent on LSRT (PCT/P126537PC00) licensed to Varian Medical, three non-patented invention (softwares) licensed to ptTheragnostic/DNAmito, Radiomics SA and Health Innovation Ventures, and three non-issued, non-licensed patents on Deep & handcrafted Radiomics (US P125078US00, PCT/NL/2020/050794, n° N2028271). He confirms that none of the above entities or funding was involved in the preparation of this paper. The remaining authors declare that the research was conducted in the absence of any commercial or financial relationships that could be construed as a potential conflict of interest.

## Publisher’s Note

All claims expressed in this article are solely those of the authors and do not necessarily represent those of their affiliated organizations, or those of the publisher, the editors and the reviewers. Any product that may be evaluated in this article, or claim that may be made by its manufacturer, is not guaranteed or endorsed by the publisher.

## Footnotes

1
http://www.fil.ion.ucl.ac.uk/spm

2
https://www.applied-statistics.de/lst.html

3
https://github.com/CyclotronResearchCentre/USwLesion
