# Supplementary material for: Exploratory Radiomic Analysis of Conventional vs. Quantitative Brain MRI: Toward Automatic Diagnosis of Early Multiple Sclerosis
Source: Front Neurosci. 2021 Aug 5;15:679941. doi: 10.3389/fnins.2021.679941 (PMC8374240; doi:10.3389/fnins.2021.679941)
Supplement: Supplementary file 1 [file Table_1.DOCX]

Supplementary Material

*Supplementary Table 1 – Multi-echo 3D FLASH acquisition parameters (Lommers et al.,* *2019)*

|  | Magnetom ALLEGRA | Magnetom PRISMA |
| --- | --- | --- |
| PDw | | |
| TR | 23.7 [ms] | 24.5 [ms] |
| Flip angle | 6° | 6° |
| Bipolar gradient echoes/TE | 6/TE 2.2-14.7 [ms] | 8/TE 2.34-18.72 [ms] |
| T1w | | |
| TR | 18.7 [ms] | 24.5 [ms] |
| Flip angle | 20° | 21° |
| Bipolar gradient echoes/TE | 6/TE 2.2-14.7 [ms] | 8/TE 2.34-18.72 [ms] |
| MTw | | |
| TR | 23.7 [ms] | 24.5 [ms] |
| Flip angle | 6° | 6° |
| Bipolar gradient echoes/TE | 6/TE 2.2-14.7 [ms] | 6/TE 2.34-14.04 [ms] |
| Bandwidth | 425 [Hz/Px] | 465 [Hz/Px] |
| Off-resonance Gaussian MT pulse | FA: 215°  Frequency offset: 2[kHz] | FA: 220°  Frequency offset: 2[kHz] |

*Supplementary Table 2 – Demographic data (Lommers et al., 2019)*

|  | All Patients  (n=36) | RRMS  (n=15, 41.6%) | SPMS  (n=7, 18.4%) | PPMS  (n=14, 38.8%) | HC  (n=36) |
| --- | --- | --- | --- | --- | --- |
| Age  y, mean (SD) | 45.69  (11.85) | 36.53  (9.32) ° | 50.14  (6.46) | 53.28  (9.78) | 45.86  (12.45) |
| Sex ratio  (F/M) | 21/15 | 9/6 | 7/0 | 5/9 | 20/16 |
| Disease duration  y, median (range) | 13  (0,5 to 35) | 6  (0.5 to 28) | 21  (9 to 35) | 12.5  (2 to 35) | N/A |
| Baseline EDSS  median (range) | 4  (1 to 6.5) | 2  (1 to 5.5) ° | 4  (3.5 to 6) | 4.75  (3 to 6.5) | N/A |
| Number of relapses median (range) | 1.5  (0 to 10) | 4  (1 to 6) | 10  (2 to 10) | 0 | N/A |

°Difference between RRMS and PMS statistically significant (alpha value of 0.05).

Abbreviations: RRMS = relapsing-remitting multiple sclerosis, SPMS = secondary progressive multiple sclerosis, PPMS = primary progressive multiple sclerosis, PMS = progressive multiple sclerosis (SPMS and PPMS), HC = healthy controls.

*Supplementary Table 3 – Quantitative MR parameters (Lommers et al., 2019)*

|  | **RRMS** | **PMS** | **HC** |
| --- | --- | --- | --- |
| **Scanner 1/ Scanner 2** | 11/4 | 15/6 | 11/25 |
| **Volumetric Data, %, mean (SD)** | | | |
| BPF | 84.99 (2.02) | 83.67 (2.47) * | 85.39 (1.75) |
| GMF | 51.06 (2.11) | 48.49 (3.16) * | 52.76 (1.99) |
| Lesion F | 1.21 (0.98) | 2.26 (1.50) ° | N/A |
| **Median MPM values, mean (SD)** | | | |
| ***MT (p.u)*** | | | |
| NACGM | 0.71 (0.09) ** | 0.68 (0.09) * | 0.82 (0.09) |
| NADGM | 0.91 (0.12) | 0.83 (0.10) * | 0.98 (1.13) |
| NAWM | 1.49 (0.18) ** | 1.45 (0.16) * | 1.68 (0.14) |
| Lesion | 0.96 (0.26) | 0.89 (0.24) | N/A |
| ***R1 (Hz)*** | | | |
| NACGM | 0.62 (0.02) ** | 0.61 (0.03) * | 0.64 (0.02) |
| NADGM | 0.75 (0.05) | 0.75 (0.05) | 0.77 (0.06) |
| NAWM | 0.99 (0.05) ** | 0.99 (0.05) * | 1.04 (0.03) |
| Lesion | 0.78 (0.12) | 0.75 (0.10) | N/A |
| ***R2* (Hz)*** | | | |
| NACGM | 15.20 (1.19) ** | 15.35 (1.17) * | 16.62 (1.02) |
| NADGM | 20.74 (2.63) | 22.46 (2.94) | 22.04 (3.10) |
| NAWM | 20.08 (1.34) ** | 20.24 (1.27) * | 21.60 (1.03) |
| Lesion | 15.03 (2.23) | 14.51 (2.30) | N/A |

° Difference between RRMS and PMS statistically significant (alpha value of 0.05).

* Difference between PMS and HC statistically significant (alpha value of 0.05).

** Difference between RRMS and HC statistically significant (alpha value of 0.05).

Abbreviations: BPF = brain parenchymal fraction, GMF = grey matter fraction, Lesion F = lesion fraction, NACGM = normal appearing cortical grey matter, NADGM = normal appearing deep grey matter, NAWM = normal appearing white matter, N/A = not applicable.

*Supplementary Table 4 – The p-values for comparison of age and sex distributions in HCS and MSP, and development and validation groups (age – Mann-Whitney test, sex, scanner, and outcome – Fisher’s exact test)*

| Groups | Age | Sex |
| --- | --- | --- |
| HCS vs MSP in DS1 | 0.90 | 1.00 |
| HCS vs MSP in DS2 + DS3 | **<0.01** | 1.00 |
| DS1 vs DS2 + DS3 | 0.73 | 0.40 |

*Supplementary Table 5 – List of extracted radiomic features per ROI (Van Griethuysen et al., 2017)*

| Feature class | Feature names |
| --- | --- |
| First Order Statistics | 10Percentile, 90Percentile, Energy, Entropy, Interquartile Range, Kurtosis, Maximum, Mean Absolute Deviation, Mean, Median, Minimum, Range, Robust Mean Absolute Deviation, Root Mean Squared, Skewness, Total Energy, Uniformity, Variance |
| Shape Based | Elongation, Flatness, Least Axis Length, Major Axis Length, Maximum 2D Diameter Column, Maximum 2D Diameter Row, Maximum 2D Diameter Slice, Maximum 3D Diameter, Mesh Volume, Minor Axis Length, Sphericity, Surface Area, Surface Volume Ratio, Voxel Volume |
| GLCM | Autocorrelation, Cluster Prominence, Cluster Shade, Cluster Tendency, Contrast, Correlation, Difference Average, Difference Entropy, Difference Variance, Id, Idm, Idmn, Idn, Imc1, Imc2, Inverse Variance, Joint Average, Joint Energy, Joint Entropy, MCC, Maximum Probability, Sum Average, Sum Entropy, Sum Squares |
| GLRLM | Gray Level Non Uniformity, Gray Level Non Uniformity Normalized, Gray Level Variance, High Gray Level Run Emphasis, Long Run Emphasis, Long Run High Gray Level Emphasis, Long Run Low Gray Level Emphasis, Low Gray Level Run Emphasis, Run Entropy, Run Length Non Uniformity, Run Length Non Uniformity Normalized, Run Percentage, Run Variance, Short Run Emphasis, Short Run High Gray Level Emphasis, Short Run Low Gray Level Emphasis |
| GLSZM | Gray Level Non Uniformity, Gray Level Non Uniformity Normalized, Gray Level Variance, High Gray Level Zone Emphasis, Large Area Emphasis, Large Area High Gray Level Emphasis, Large Area Low Gray Level Emphasis, Low Gray Level Zone Emphasis, Size Zone Non Uniformity, Size Zone Non Uniformity Normalized, Small Area Emphasis, Small Area High Gray Level Emphasis, Small Area Low Gray Level Emphasis, Zone Entropy, Zone Percentage, Zone Variance |
| NGTDM | Dependence Entropy, Dependence Non Uniformity, Dependence Non Uniformity Normalized, Dependence Variance, Gray Level Non Uniformity, Gray Level Variance, High Gray Level Emphasis, Large Dependence Emphasis, Large Dependence High Gray Level Emphasis, Large Dependence Low Gray Level Emphasis, Low Gray Level Emphasis, Small Dependence Emphasis, Small Dependence High Gray Level Emphasis, Small Dependence Low Gray Level Emphasis |
| GLDM | Busyness, Coarseness, Complexity, Contrast, Strength |

*Supplementary Table 6 – Subjects distribution in training and testing subsets of DATASET 1 with p-values for the corresponding distribution comparison statistical tests*

| Subset | Training | Testing | p-value* |
| --- | --- | --- | --- |
| Size | 57 | 15 |  |
| Age (mean, min, max) | 45.8, 21, 65 | 45.6, 26, 62 | 0.99 |
| Sex (M, F) | 24, 33 | 7, 8 | 0.77 |
| Scanner (1, 2) | 29, 28 | 8, 7 | 1.00 |
| Outcome (0, 1) | 29, 28 | 7, 8 | 1.00 |

* age – Mann-Whitney test, sex, scanner, and outcome – Fisher’s exact test

*Supplementary Table 7 – Number of features per set, kept after feature selection steps*

|  | ROI | T1w | PD | MT | R1 | R2s | qMRI_comb_ |
| --- | --- | --- | --- | --- | --- | --- | --- |
| Original features set | WM | 93 | 93 | 93 | 93 | 93 | 372 |
|  | NAWM | 93 | 93 | 93 | 93 | 93 | 372 |
|  | GM | 93 | 93 | 93 | 93 | 93 | 372 |
| Low variance step | WM | 85 | 90 | 86 | 85 | 90 | 351 |
|  | NAWM | 85 | 85 | 85 | 85 | 85 | 340 |
|  | GM | 91 | 86 | 90 | 88 | 92 | 356 |
| High correlation step | WM | 21 | 19 | 23 | 20 | 18 | 69 |
|  | NAWM | 22 | 19 | 20 | 22 | 17 | 73 |
|  | GM | 20 | 18 | 18 | 23 | 19 | 70 |
| Final features vector | WM | 3 | 3 | 3 | 3 | 3 | 3 |
|  | NAWM | 3 | 3 | 3 | 3 | 3 | 3 |
|  | GM | 3 | 3 | 3 | 3 | 3 | 3 |

*Supplementary Table 8 – List of the selected features*

| Image | WM | NAWM | GM |
| --- | --- | --- | --- |
| T1w | glcm_ClusterShade  firstorder_Range  glszm_SmallAreaEmphasis | glcm_ClusterShade  firstorder_Variance  firstorder_90Percentile | firstorder_10Percentile  firstorder_Skewness  firstorder_Kurtosis |
| PD | firstorder_Skewness  gldm_LargeDependenceHighGrayLevelEmphasis  glcm_MCC | gldm_LargeDependenceHighGrayLevelEmphasis  glcm_ClusterProminence  glcm_ClusterShade | firstorder_10Percentile  firstorder_InterquartileRange  firstorder_Skewness |
| MT | firstorder_Minimum  glcm_ClusterProminence  glcm_MCC | gldm_LargeDependenceHighGrayLevelEmphasis  ngtdm_Complexity  firstorder_TotalEnergy | gldm_SmallDependenceLowGrayLevelEmphasis  firstorder_90Percentile  glszm_LargeAreaHighGrayLevelEmphasis |
| R1 | firstorder_Kurtosis  glcm_ClusterShade  glcm_MCC | firstorder_10Percentile  firstorder_Minimum  firstorder_MeanAbsoluteDeviation | firstorder_Minimum  firstorder_TotalEnergy  firstorder_10Percentile |
| R2* | glcm_ClusterShade  firstorder_Skewness  firstorder_Minimum | glcm_Imc2  gldm_LargeDependenceHighGrayLevelEmphasis  gldm_DependenceEntropy | gldm_LowGrayLevelEmphasis  firstorder_Median  firstorder_TotalEnergy |
| qMRI_comb_ | PD_gldm_LargeDependenceHighGrayLevelEmphasis  R1_firstorder_Kurtosis  MT_firstorder_Minimum | R1_firstorder_Minimum  MT_firstorder_Kurtosis  PD_glcm_ClusterProminence | R1_firstorder_Minimum  R1_firstorder_TotalEnergy  MT_gldm_SmallDependenceLowGrayLevelEmphasis |

*Supplementary Figure 1 – Absolute values of Spearman correlation coefficients of the selected features with age* $\left| \boldsymbol{r}_{\boldsymbol{age}} \right|$ *and ROI volume* $\left| \boldsymbol{r}_{\boldsymbol{volume}} \right|$


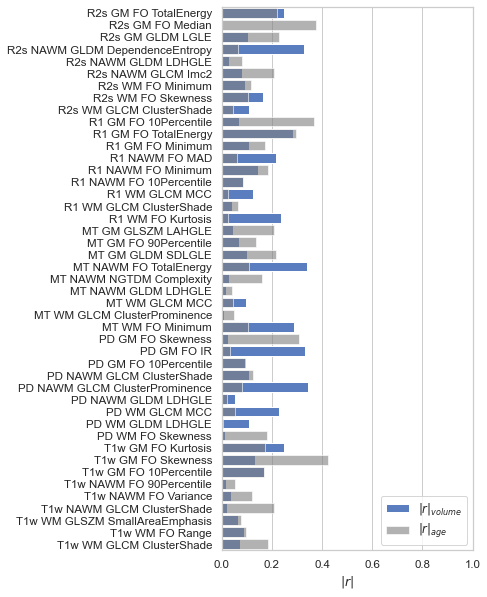


*Supplementary Table 9 – Univariate ROC AUC scores and correlation with the selected features of the quantitative values of the qMRI sequences (correlation coefficients with the absolute value* $\left| \boldsymbol{r}_{\boldsymbol{S}} \right|\boldsymbol{>0.85}$ *are highlighted with the bold font)*

| Image type | Univariate ROC AUC | $\left\vert\boldsymbol{r}_{\boldsymbol{S}}^{\boldsymbol{1}} \right\vert$ | $\left\vert\boldsymbol{r}_{\boldsymbol{S}}^{\boldsymbol{2}} \right\vert$ | $\left\vert\boldsymbol{r}_{\boldsymbol{S}}^{\boldsymbol{3}} \right\vert$ |
| --- | --- | --- | --- | --- |
| WM |  |  |  |  |
| T1w | 0.52 | 0.13 | 0.23 | 0.10 |
| PD | 0.84 | 0.77 | 0.29 | 0.48 |
| MT | 0.86 | 0.62 | 0.60 | 0.39 |
| R1 | 0.84 | 0.48 | 0.56 | 0.37 |
| R2* | 0.83 | 0.43 | 0.66 | 0.71 |
| NAWM |  |  |  |  |
| T1w | 0.66 | 0.14 | 0.02 | 0.68 |
| PD | 0.65 | 0.16 | 0.27 | 0.70 |
| MT | 0.84 | 0.77 | 0.58 | 0.93 |
| R1 | 0.81 | **0.89** | 0.50 | 0.17 |
| R2* | 0.80 | 0.74 | 0.71 | 0.47 |
| GM |  |  |  |  |
| T1w | 0.68 | 0.56 | 0.49 | 0.39 |
| PD | 0.64 | 0.69 | 0.18 | 0.26 |
| MT | 0.84 | 0.78 | **0.94** | 0.75 |
| R1 | 0.74 | 0.45 | 0.74 | 0.79 |
| R2* | 0.76 | 0.36 | **0.98** | 0.85 |

$r_{S}^{i}$ ***–*** Spearman correlation coefficient of the mean qMRI value with the *i*-th selected feature in the given image type and ROI

*Supplementary Table 10 – Delong test p-values for ROC AUCs, obtained with different ML methods (RFC, SV, LR) (Bonferroni correction) (p-values for significant differences are highlighted with bold font)*

|  | RFC vs SV | RFC vs LR | LR vs SV |
| --- | --- | --- | --- |
| WM |  |  |  |
| T1w | <**0.01** | <**0.01** | <**0.01** |
| PD | <**0.01** | <**0.01** | <**0.01** |
| MT | 1 | 1 | 1 |
| R1 | <**0.01** | <**0.01** | <**0.01** |
| R2* | <**0.01** | <**0.01** | <**0.01** |
| qMRI_comb_ | <**0.01** | 1 | <**0.01** |
| NAWM |  |  |  |
| T1w | <**0.01** | <**0.01** | <**0.01** |
| PD | <**0.01** | <**0.01** | <**0.01** |
| MT | <**0.01** | <**0.01** | <**0.01** |
| R1 | <**0.01** | <**0.01** | 1 |
| R2* | <**0.01** | <**0.01** | <**0.01** |
| qMRI_comb_ | <**0.01** | <**0.01** | <**0.01** |
| GM |  |  |  |
| T1w | <**0.01** | <**0.01** | <**0.01** |
| PD | 0.30 | <**0.01** | <**0.01** |
| MT | <**0.01** | <**0.01** | <**0.01** |
| R1 | <**0.01** | <**0.01** | <**0.01** |
| R2* | <**0.01** | <**0.01** | <**0.01** |
| qMRI_comb_ | <**0.01** | <**0.01** | <**0.01** |

*Supplementary Table 11 – Performance metrics comparison on testing data for different machine learning methods showing the median (90% CI) for each image and tissue type (ROI) (median values above 0.7 for all the performance metrics for the same model are highlighted with bold font)*

| Model | | AUC | | | | Sensitivity | | | Specificity |
| --- | --- | --- | --- | --- | --- | --- | --- | --- | --- |
| WM T1w | | | | | | | | | |
| RFC | | **0.93** (0.88, 0.98) | | | | **1.00** (1.00, 1.00) | | | **0.86** (0.76, 0.95) |
| SVM | | **0.79** (0.72, 0.87) | | | | **0.88** (0.78, 0.94) | | | **0.72** (0.59, 0.82) |
| LR | | **0.74** (0.66, 0.82) | | | | **0.76** (0.67, 0.86) | | | **0.72** (0.59, 0.82) |
| WM PD | | |  | | | |  | | |
| RFC | | 0.64 (0.58, 0.71) | | | | 1.00 (1.00, 1.00) | | | 0.28 (0.17, 0.42) |
| SVM | | 0.64 (0.58, 0.71) | | | | 1.00 (1.00, 1.00) | | | 0.28 (0.17, 0.42) |
| LR | | 0.64 (0.58, 0.71) | | | | 1.00 (1.00, 1.00) | | | 0.28 (0.17, 0.42) |
| WM MT | | |  | | | |  | | |
| RFC | | **0.93** (0.88, 0.97) | | | | **1.00** (1.00, 1.00) | | | **0.86** (0.78, 0.92) |
| SVM | | **1.00** (1.00, 1.00) | | | | **1.00** (1.00, 1.00) | | | **1.00** (1.00, 1.00) |
| LR | | **1.00** (1.00, 1.00) | | | | **1.00** (1.00, 1.00) | | | **1.00** (1.00, 1.00) |
| WM R1 | | |  | | | |  | | |
| RFC | | 0.76 (0.69, 0.83) | | | | 0.51 (0.38, 0.66) | | | 1.00 (1.00, 1.00) |
| SVM | | 0.76 (0.69, 0.82) | | | | 0.52 (0.38, 0.64) | | | 1.00 (1.00, 1.00) |
| LR | | 0.82 (0.76, 0.88) | | | | 0.64 (0.52, 0.75) | | | 1.00 (1.00, 1.00) |
| WM R2* | | |  | | | |  | | |
| RFC | | 0.66 (0.57, 0.73) | | | | 0.88 (0.79, 0.95) | | | 0.43 (0.28, 0.57) |
| SVM | | 0.68 (0.57, 0.77) | | | | 0.64 (0.50, 0.74) | | | 0.72 (0.58, 0.84) |
| LR | | **0.73** (0.63, 0.83) | | | | **0.76** (0.62, 0.86) | | | **0.72** (0.58, 0.84) |
| WM qMRIcomb | | |  | | | |  | | |
| RFC | | **0.94** (0.90, 0.98) | | | | **0.88** (0.80, 0.96) | | | **1.00** (1.00, 1.00) |
| SVM | | **0.86** (0.79, 0.92) | | | | **0.88** (0.74, 0.97) | | | **0.86** (0.77, 0.94) |
| LR | | **0.93** (0.88, 0.97) | | | | **1.00** (1.00, 1.00) | | | **0.86** (0.77, 0.94) |
| NAWM T1w | | |  | | | |  | | |
| RFC | | **0.93** (0.88, 0.97) | | | | **1.00** (1.00, 1.00) | | | **0.86** (0.77, 0.94) |
| SVM | | 0.82 (0.75, 0.88) | | | | 0.64 (0.50, 0.76) | | | 1.00 (1.00, 1.00) |
| LR | | **0.73** (0.66, 0.82) | | | | **0.76** (0.64, 0.87) | | | **0.70** (0.59, 0.81) |
| NAWM PD | | |  | | | |  | | |
| RFC | | 0.37 (0.30, 0.44) | | | | 0.74 (0.60, 0.87) | | | 0.00 (0.00, 0.00) |
| SVM | | 0.37 (0.30, 0.44) | | | | 0.74 (0.60, 0.87) | | | 0.00 (0.00, 0.00) |
| LR | | 0.37 (0.30, 0.44) | | | | 0.74 (0.60, 0.87) | | | 0.00 (0.00, 0.00) |
| NAWM MT | | |  | | | |  | | |
| RFC | | **0.81** (0.74, 0.89) | | | | **0.76** (0.64, 0.87) | | | **0.86** (0.77, 0.94) |
| SVM | | **0.81** (0.74, 0.89) | | | | **0.76** (0.64, 0.87) | | | **0.86** (0.77, 0.94) |
| LR | | **0.81** (0.74, 0.89) | | | | **0.76** (0.64, 0.87) | | | **0.86** (0.77, 0.94) |
| NAWM R1 | | |  | | | |  | | |
| RFC | | **0.93** (0.88, 0.97) | | | | **1.00** (1.00, 1.00) | | | **0.86** (0.77, 0.94) |
| SVM | | **0.87** (0.80, 0.93) | | | | **0.88** (0.77, 0.98) | | | **0.86** (0.77, 0.94) |
| LR | | **0.87** (0.80, 0.93) | | | | **0.88** (0.77, 0.98) | | | **0.86** (0.77, 0.94) |
| NAWM R2* | | |  | | | |  | | |
| RFC | | **0.79** (0.71, 0.88) | | | | **0.88** (0.77, 0.98) | | | **0.72** (0.60, 0.81) |
| SVM | | 0.66 (0.56, 0.76) | | | | 0.76 (0.64, 0.87) | | | 0.56 (0.40, 0.72) |
| LR | | 0.66 (0.56, 0.76) | | | | 0.76 (0.64, 0.87) | | | 0.56 (0.40, 0.72) |
| NAWM qMRIcomb | | |  | | | |  | | |
| RFC | | **0.79** (0.71, 0.88) | | | | **0.88** (0.78, 0.94) | | | **0.71** (0.58, 0.84) |
| SVM | | **0.87** (0.80, 0.93) | | | | **0.88** (0.77, 0.98) | | | **0.86** (0.77, 0.94) |
| LR | | 0.74 (0.67, 0.81) | | | | 0.62 (0.48, 0.77) | | | 0.86 (0.77, 0.94) |
| GM T1w | | | |  | | | | | |
| RFC | 0.47 (0.38, 0.56) | | | | 0.24 (0.12, 0.37) | | | 0.71 (0.58, 0.84) | |
| SVM | 0.49 (0.41, 0.55) | | | | 0.12 (0.05, 0.21) | | | 0.86 (0.75, 0.94) | |
| LR | 0.41 (0.32, 0.52) | | | | 0.26 (0.16, 0.40) | | | 0.56 (0.43, 0.71) | |
| GM PD | | | |  | | | | | |
| RFC | 0.65 (0.57, 0.75) | | | | 0.88 (0.77, 0.98) | | | 0.43 (0.29, 0.54) | |
| SVM | 0.76 (0.69, 0.83) | | | | 0.51 (0.38, 0.66) | | | 1.00 (1.00, 1.00) | |
| LR | 0.69 (0.61, 0.79) | | | | 0.51 (0.38, 0.66) | | | 0.86 (0.77, 0.94) | |
| GM MT | | | |  | | | | | |
| RFC | 0.82 (0.75, 0.87) | | | | 0.64 (0.50, 0.74) | | | 1.00 (1.00, 1.00) | |
| SVM | **0.88** (0.82, 0.94) | | | | **0.76** (0.64, 0.87) | | | **1.00** (1.00, 1.00) | |
| LR | **0.88** (0.82, 0.94) | | | | **0.76** (0.64, 0.87) | | | **1.00** (1.00, 1.00) | |
| GM R1 | | | |  | | | | | |
| RFC | 0.74 (0.66, 0.81) | | | | 0.62 (0.52, 0.75) | | | 0.84 (0.77, 0.95) | |
| SVM | **0.81** (0.73, 0.88) | | | | **0.76** (0.64, 0.87) | | | **0.84** (0.77, 0.95) | |
| LR | 0.82 (0.75, 0.87) | | | | 0.64 (0.50, 0.74) | | | 1.00 (1.00, 1.00) | |
| GM R2* |  | | | |  | | |  | |
| RFC | 0.58 (0.48, 0.68) | | | | 0.76 (0.64, 0.87) | | | 0.43 (0.29, 0.54) | |
| SVM | **0.73** (0.65, 0.83) | | | | **0.76** (0.64, 0.87) | | | **0.71** (0.58, 0.84) | |
| LR | **0.73** (0.65, 0.83) | | | | **0.76** (0.64, 0.87) | | | **0.71** (0.58, 0.84) | |
| GM qMRIcomb | | | | |  | | |  | |
| RFC | 0.74 (0.66, 0.81) | | | | 0.62 (0.52, 0.75) | | | 0.84 (0.77, 0.95) | |
| SVM | **0.81** (0.73, 0.88) | | | | **0.76** (0.64, 0.87) | | | **0.84** (0.77, 0.95) | |
| LR | **0.81** (0.73, 0.88) | | | | **0.76** (0.64, 0.87) | | | **0.84** (0.77, 0.95) | |

*Supplementary Table 12 – LR coefficients for the trained models*

| ROI | Image | Coefficients |
| --- | --- | --- |
| WM | T1w | -1.47718153, 2.59667219, -0.85246602 |
|  | PD | 2.53718314, -3.22015231, 2.7345865 |
|  | MT | -2.80174761, 1.4198698 , 2.46118085 |
|  | R1 | 2.6844983 , -2.31225968, 2.51263994 |
|  | R2* | -1.36964725, -2.18103749, -0.315707 |
|  | qMRI_comb_ | -2.57664498, 2.55077121, -2.39050481 |
| NAWM | T1w | -0.90383097, 1.5330599 , 2.15768717 |
|  | PD | -2.9891113 , 2.34756699, 1.91013323 |
|  | MT | -1.87930192, 1.60219702, -1.15724868 |
|  | R1 | -3.03645147, -0.69411281, 2.2771456 |
|  | R2* | -2.01972909, -0.94084113, -0.65891473 |
|  | qMRI_comb_ | -2.11214775, -1.47300678, 3.23379527 |
| GM | T1w | -2.7500275 , 0.79583978, -0.78646721 |
|  | PD | -0.89519434, 1.42562508, 0.15981217 |
|  | MT | 2.32695847, -1.85744863, -0.01174804 |
|  | R1 | -0.94392645, -1.91957481, -1.33275998 |
|  | R2* | 2.10804906, -1.45236701, -1.60858426 |
|  | qMRI_comb_ | -1.05899899, -2.29378015, 2.70636515 |

*Supplementary Table 13 - Delong test p-values for LR ROC AUCs for each image type within the same ROI (Bonferroni correction) (p-values for significant differences are highlighted with bold font)*

|  | WM | NAWM | GM |
| --- | --- | --- | --- |
| T1w vs PD | <**0.01** | <**0.01** | <**0.01** |
| T1w vs MT | <**0.01** | <**0.01** | <**0.01** |
| T1w vs R1 | <**0.01** | <**0.01** | <**0.01** |
| T1w vs R2* | <**0.01** | <**0.01** | <**0.01** |
| T1w vs qMRI_comb_ | <**0.01** | <**0.01** | <**0.01** |
| PD vs MT | <**0.01** | <**0.01** | <**0.01** |
| PD vs R1 | <**0.01** | <**0.01** | <**0.01** |
| PD vs R2* | <**0.01** | <**0.01** | <**0.01** |
| PD vs qMRI_comb_ | <**0.01** | <**0.01** | <**0.01** |
| MT vs R1 | 1 | <**0.01** | 0.87 |
| MT vs R2* | <**0.01** | <**0.01** | <**0.01** |
| MT vs qMRI_comb_ | 1 | <**0.01** | <**0.01** |
| R1 vs R2* | <**0.01** | <**0.01** | <**0.01** |
| R1 vs qMRI_comb_ | 1 | <**0.01** | <**0.01** |
| R2* vs qMRI_comb_ | <**0.01** | 0.35 | 0.95 |

*Supplementary Table 14 - Classification models performance on testing data on permutation test: median (90% CI)*

| ROI | Image | AUC | Sensitivity | Specificity |
| --- | --- | --- | --- | --- |
| WM | T1w | 0.56 (0.48, 0.63) | 0.26 (0.15, 0.39) | 0.88 (0.76, 0.94) |
|  | PD | 0.60 (0.51, 0.69) | 0.62 (0.49, 0.75) | 0.56 (0.45, 0.70) |
|  | MT | 0.48 (0.40, 0.58) | 0.38 (0.28, 0.50) | 0.58 (0.45, 0.71) |
|  | R1 | 0.35 (0.29, 0.43) | 0.14 (0.06, 0.24) | 0.58 (0.45, 0.72) |
|  | R2* | 0.59 (0.51, 0.71) | 0.75 (0.64, 0.87) | 0.44 (0.31, 0.58) |
|  | qMRI_comb_ | 0.45 (0.35, 0.52) | 0.62 (0.46, 0.73) | 0.28 (0.18, 0.38) |
| NAWM | T1w | 0.41 (0.31, 0.52) | 0.26 (0.15, 0.39) | 0.56 (0.40, 0.72) |
|  | PD | 0.49 (0.43, 0.56) | 0.12 (0.06, 0.22) | 0.86 (0.76, 0.95) |
|  | MT | 0.60 (0.49, 0.68) | 0.48 (0.34, 0.62) | 0.71 (0.58, 0.80) |
|  | R1 | 0.47 (0.37, 0.58) | 0.50 (0.38, 0.64) | 0.44 (0.31, 0.58) |
|  | R2* | 0.58 (0.50, 0.66) | 0.88 (0.78, 0.94) | 0.30 (0.16, 0.40) |
|  | qMRI_comb_ | 0.43 (0.37, 0.47) | 0.00 (0.00, 0.00) | 0.86 (0.75, 0.94) |
| GM | T1w | 0.67 (0.57, 0.76) | 0.76 (0.63, 0.88) | 0.56 (0.43, 0.72) |
|  | PD | 0.47 (0.38, 0.57) | 0.50 (0.38, 0.62) | 0.42 (0.27, 0.59) |
|  | MT | 0.48 (0.37, 0.57) | 0.36 (0.22, 0.49) | 0.58 (0.45, 0.71) |
|  | R1 | 0.52 (0.45, 0.61) | 0.62 (0.46, 0.74) | 0.42 (0.32, 0.56) |
|  | R2* | 0.63 (0.58, 0.70) | 0.26 (0.16, 0.40) | 1.00 (1.00, 1.00) |
|  | qMRI_comb_ | 0.39 (0.30, 0.52) | 0.38 (0.22, 0.53) | 0.42 (0.28, 0.56) |

*Supplementary Table 15 - Permutation test p-values for LR ROC AUCs for the original and randomized outcomes testing models within the same ROI and image type (p-values for significant differences,* $\boldsymbol{p\leq0.01}$*, are highlighted with bold font)*

|  | WM | NAWM | GM |
| --- | --- | --- | --- |
| T1w | **0.00** | **0.00** | 1 |
| PD | 0.16 | 0.96 | **0.00** |
| MT | **0.00** | **0.00** | **0.00** |
| R1 | **0.00** | **0.00** | **0.00** |
| R2* | 0.05 | 0.10 | **0.03** |
| qMRI_comb_ | **0.00** | **0.00** | **0.00** |

*Supplementary Table 16 – Univariate ROC AUC scores for the demographic and clinical variables of the DS1*

| Variable | Univariate ROC AUC |
| --- | --- |
| Age | 0.50 |
| Brain parenchymal fraction | 0.65 |
| GM fraction | 0.83 |
| WM fraction | 0.70 |
| Motor tests Z-score° | 0.84 |
| Cognitive test Z-score* | 0.70 |

° Motor tests Z-score = [Z_T25FW_+Z_9-HPT dominant hand_+Z_9-HPT non dominant hand_]/3

* Cognitive test Z-score = [Z_SDMT_+ Z_CVLT_]/2

Abbreviations: T25FW – Time 25-Foot Walk, 9-HPT – 9-Hole Peg Test (on both hands), SDMT – oral Symbol Digit Modalities test, CVLT – California Verbal Learning Test (five recalls)

Patients with MS were scored by a qualified MS specialist (ELo) on motor and cognitive tests. Z-scores for T25FW, 9-HPT, SDMT and CVLT were standardized to HC summary statistics and transformed to make improvement a positive number.

*Supplementary Table 17 - Radiomics quality score table: criteria, the maximum amount of points that can be acquired (or maximum points that can be deducted) and the points calculated in this study*

|  | Criteria | Maximum points possible | Points |
| --- | --- | --- | --- |
| 1 | Image protocol quality - well-documented image protocols (for example, contrast, slice thickness, energy, etc.) and/or usage of public image protocols allow reproducibility/replicability | + 1 (if protocols are well-documented) + 1 (if public protocol is used) | 0 |
| 2 | Multiple segmentations - possible actions are: segmentation by different physicians/algorithms/software, perturbing segmentations by (random) noise, segmentation at different breathing cycles. Analyse feature robustness to segmentation variabilities | 1 | 0 |
| 3 | Phantom study on all scanners - detect inter-scanner differences and vendor-dependent features. Analyse feature robustness to these sources of variability | 1 | 0 |
| 4 | Imaging at multiple time points - collect images of individuals at additional time points. Analyse feature robustness to temporal variabilities (for example, organ movement, organ expansion/shrinkage) | 1 | 0 |
| 5 | Feature reduction or adjustment for multiple testing - decreases the risk of overfitting. Overfitting is inevitable if the number of features exceeds the number of samples. Consider feature robustness when selecting features | − 3 (if neither measure is implemented) + 3 (if either measure is implemented) | 3 |
| 6 | Multivariable analysis with non radiomics features (for example, EGFR mutation) - is expected to provide a more holistic model. Permits correlating/inferencing between radiomics and non radiomics features | 1 | 1 |
| 7 | Detect and discuss biological correlates - demonstration of phenotypic differences (possibly associated with underlying gene–protein expression patterns) deepens understanding of radiomics and biology | 1 | 0 |
| 8 | Cut-off analyses - determine risk groups by either the median, a previously published cut-off or report a continuous risk variable. Reduces the risk of reporting overly optimistic results | 1 | 0 |
| 9 | Discrimination statistics - report discrimination statistics (for example, C-statistic, ROC curve, AUC) and their statistical significance (for example, p-values, confidence intervals). One can also apply resampling method (for example, bootstrapping, cross-validation) | + 1 (if a discrimination statistic and its statistical significance are reported) + 1 (if a resampling method technique is also applied) | 2 |
| 10 | Calibration statistics - report calibration statistics (for example, Calibration-in-the-large/slope, calibration plots) and their statistical significance (for example, *P*-values, confidence intervals). One can also apply resampling method (for example, bootstrapping, cross-validation) | + 1 (if a calibration statistic and its statistical significance are reported) + 1 (if a resampling method technique is also applied) | 1 |
| 11 | Prospective study registered in a trial database - provides the highest level of evidence supporting the clinical validity and usefulness of the radiomics biomarker | + 7 (for prospective validation of a radiomics signature in an appropriate trial) | 0 |
| 12 | Validation - the validation is performed without retraining and without adaptation of the cut-off value, provides crucial information with regard to credible clinical performance | - 5 (if validation is missing) + 2 (if validation is based on a dataset from the same institute) + 3 (if validation is based on a dataset from another institute) + 4 (if validation is based on two datasets from two distinct institutes) + 4 (if the study validates a previously published signature) + 5 (if validation is based on three or more datasets from distinct institutes) | 4 |
| 13 | Comparison to 'gold standard' - assess the extent to which the model agrees with/is superior to the current 'gold standard' method (for example, TNM-staging for survival prediction). This comparison shows the added value of radiomics | 2 | 0 |
| 14 | Potential clinical utility - report on the current and potential application of the model in a clinical setting (for example, decision curve analysis). | 2 | 2 |
| 15 | Cost-effectiveness analysis - report on the cost-effectiveness of the clinical application (for example, QALYs generated) | 1 | 0 |
| 16 | Open science and data - make code and data publicly available. Open science facilitates knowledge transfer and reproducibility of the study | + 1 (if scans are open source) + 1 (if region of interest segmentations are open source) + 1 (if code is open source) + 1 (if radiomics features are calculated on a set of representative ROIs and the calculated features and representative ROIs are open source) | 1 |
|  | **Total score:** | **36** | **14** |

*Supplementary Table 18 - TRIPOD adherence data extraction checklist: Prediction Model Development and Validation*

|  | **Y=yes; N=no; R=referenced; NA=not applicable** | **Development [D]** | **External validation [V]** | **Combined Development & External validation [D+V]** |
| --- | --- | --- | --- | --- |
| **Title and abstract** | |  |  |  |
| **1** | **Identify the study as developing and/or validating a multivariable prediction model, the target population, and the outcome to be predicted.** | 0 | 0 | **0** |
| i | The words developing/development, validation/validating, incremental/added value (or synonyms) are reported in the title | N | N | N |
| ii | The words prediction, risk prediction, prediction model, risk models, prognostic models, prognostic indices, risk scores (or synonyms) are reported in the title | N | N | N |
| iii | The target population is reported in the title | Y | Y | Y |
| iv | The outcome to be predicted is reported in the title | N | N | N |
| **2** | **Provide a summary of objectives, study design, setting, participants, sample size, predictors, outcome, statistical analysis, results, and conclusions.** | 0 | 0 | **0** |
| i | The objectives are reported in the abstract | Y | Y | Y |
| ii | Sources of data are reported in the abstract *E.g. Prospective cohort, registry data, RCT data.* | Y | Y | Y |
| iii | The setting is reported in the abstract *E.g. Primary care, secondary care, general population, adult care, or paediatric care. The setting should be reported for both the development and validation datasets, if applicable.* | N | N | N |
| iv | A general definition of the study participants is reported in the abstract *E.g. patients with suspicion of certain disease, patients with a specific disease, or general eligibility criteria.* | Y | Y | Y |
| v | The overall sample size is reported in the abstract | Y | Y | Y |
| vi | The number of events (or % outcome together with overall sample size) is reported in the abstract *If a continuous outcome was studied, score Not applicable (NA)*. | Y | Y | Y |
| vii | Predictors included in the final model are reported in the abstract. For validation studies of well-known models, at least the name/acronym of the validated model is reported *Broad descriptions are sufficient, e.g. ‘all information from patient history and physical examination’. Check in the main text whether all predictors of the final model are indeed reported in the abstract.* | N | N | N |
| viii | The outcome is reported in the abstract | Y | Y | Y |
| ix | Statistical methods are described in the abstract *For model development, at least the type of statistical model should be reported. For validation studies a quote like “model’s discrimination and calibration was assessed” is considered adequate. If done, methods of updating should be reported.* | Y | Y | Y |
| x | Results for model discrimination are reported in the abstract *This should be reported separately for development and validation if a study includes both development and validation.* | N | N | N |
| xi | Results for model calibration are reported in the abstract *This should be reported separately for development and validation if a study includes both development and validation.* | N | N | N |
| xii | Conclusions are reported in the abstract *In publications addressing both model development and validation, there is no need for separate conclusions for both; one conclusion is sufficient.* | Y | Y | Y |
| **3a** | **Explain the medical context (including whether diagnostic or prognostic) and rationale for developing or validating the multivariable prediction model, including references to existing models.** | 1 | 1 | **1** |
| i | The background and rationale are presented | Y | Y | Y |
| ii | Reference to existing models is included (or stated that there are no existing models) | Y | Y | Y |
| **3b** | **Specify the objectives, including whether the study describes the development or validation of the model or both.** | 1 | 1 | **1** |
| i | It is stated whether the study describes development and/or validation and/or incremental (added) value | Y | Y | Y |
| **Methods** | |  |  |  |
| **4a** | **Describe the study design or source of data (e.g., randomized trial, cohort, or registry data), separately for the development and validation data sets, if applicable.** | 1 | 1 | **1** |
| i | The study design/source of data is described *E.g. Prospectively designed, existing cohort, existing RCT, registry/medical records, case control, case series. This needs to be explicitly reported; reference to this information in another article alone is insufficient.* | Y | Y | Y |
| **4b** | **Specify the key study dates, including start of accrual; end of accrual; and, if applicable, end of follow-up.** | 1 | 0 | **0** |
| i | The starting date of accrual is reported | Y | N | N |
| ii | The end date of accrual is reported | Y | Y | Y |
| iii | The length of follow-up and prediction horizon/time frame are reported, if applicable *E.g. “Patients were followed from baseline for 10 years“ and “10-year prediction of…”; notably for prognostic studies with long term follow-up. If this is not applicable for an article (i.e. diagnostic study or no follow-up), then score Not applicable (NA).* | NA | NA | NA |
| **5a** | **Specify key elements of the study setting (e.g., primary care, secondary care, general population) including number and location of centres.** | 0 | 0 | **0** |
| i | The study setting is reported (e.g. primary care, secondary care, general population) *E.g.: ‘surgery for endometrial cancer patients’ is considered to be enough information about the study setting.* | N | N | N |
| ii | The number of centres involved is reported *If the number is not reported explicitly, but can be concluded from the name of the centre/centres, or if clearly a single centre study, score Yes.* | Y | Y | Y |
| iii | The geographical location (at least country) of centres involved is reported *If no geographical location is specified, but the location can be concluded from the name of the centre(s), score Yes.* | Y | Y | Y |
| **5b** | **Describe eligibility criteria for participants.** | 1 | 1 | **1** |
| i | In-/exclusion criteria are stated *These should explicitly be stated. Reasons for exclusion only described in a patient flow is not sufficient.* | Y | Y | Y |
| **5c** | **Give details of treatments received, if relevant.** *(i.e. notably for prognostic studies with long term follow-up)* | NA | NA | **NA** |
| i | Details of any treatments received are described  *This item is notably for prognostic modelling studies and is about treatment at baseline or during follow-up. The ‘if relevant’ judgment of treatment requires clinical knowledge and interpretation.  If you are certain that treatment was not relevant, e.g. in some diagnostic model studies, score Not applicable.* | NA | NA | NA |
| **6a** | **Clearly define the outcome that is predicted by the prediction model, including how and when assessed.** | 0 | 0 | **0** |
| i | The outcome definition is clearly presented *This should be reported separately for development and validation if a publication includes both.* | Y | Y | Y |
| ii | It is described how outcome was assessed (including all elements of any composite, for example CVD [e.g. MI, HF, stroke]). | Y | N | N |
| iii | It is described when the outcome was assessed (time point(s) since T0) | N | N | N |
| **6b** | **Report any actions to blind assessment of the outcome to be predicted.** | 0 | 0 | **0** |
| i | Actions to blind assessment of outcome to be predicted are reported *If it is clearly a non-issue (e.g. all-cause mortality or an outcome not requiring interpretation), score Yes. In all other instances, an explicit mention is expected*. | N | N | N |
| **7a** | **Clearly define all predictors used in developing or validating the multivariable prediction model, including how and when they were measured.** | 0 | 0 | **0** |
| i | All predictors are reported *For development, “all predictors” refers to all predictors that potentially could have been included in the ‘final’ model (including those considered in any univariable analyses). For validation, “all predictors” means the predictors in the model being evaluated.* | Y | Y | Y |
| ii | Predictor definitions are clearly presented | Y | Y | Y |
| iii | It is clearly described how the predictors were measured | Y | Y | Y |
| iv | It is clearly described when the predictors were measured | N | N | N |
| **7b** | **Report any actions to blind assessment of predictors for the outcome and other predictors.** | 1 | 1 | **1** |
| i | It is clearly described whether predictor assessments were blinded for outcome *For predictors for which it is clearly a non-issue (e.g. automatic blood pressure measurement, age, sex) and for instances where the predictors were clearly assessed before outcome assessment, score Yes. For all other predictors an explicit mention is expected.* | Y | Y | Y |
| ii | It is clearly described whether predictor assessments were blinded for the other predictors | Y | Y | Y |
| **8** | **Explain how the study size was arrived at.** | 1 | 1 | **1** |
| i | It is explained how the study size was arrived at *Is there any mention of sample size, e.g. whether this was done on statistical grounds or practical/logistical grounds (e.g. an existing study cohort or data set of a RCT was used)?* | Y | Y | Y |
| **9** | **Describe how missing data were handled (e.g., complete-case analysis, single imputation, multiple imputation) with details of any imputation method.** | 1 | 1 | **1** |
| i | The method for handling missing data (predictors and outcome) is mentioned *E.g. Complete case (explicit mention that individuals with missing values have been excluded), single imputation, multiple imputation, mean/median imputation. If there is no missing data, there should be an explicit mention that there is no missing data for all predictors and outcome. If so, score Yes. If it is unclear whether there is missing data (from e.g. the reported methods or results), score No. If it is clear there is missing data, but the method for handling missing data is unclear, score No.* | 1 | 1 | 1 |
| ii | If missing data were imputed, details of the software used are given *When under 9i explicit mentioning of no missing data, complete case analysis or no imputation applied, score Not applicable.* | NA | NA | NA |
| iii | If missing data were imputed, a description of which variables were included in the imputation procedure is given *When under 9i explicit mentioning of no missing data, complete case analysis or no imputation applied, score Not applicable.* | NA | NA | NA |
| iv | If multiple imputation was used, the number of imputations is reported *When under 9i explicit mentioning of no missing data, complete case analysis or no imputation applied, score Not applicable.* | NA | NA | NA |
| **10a** | **Describe how predictors were handled in the analyses.** | 1 | NA | **1** |
| i | For continuous predictors it is described whether they were modelled as linear, nonlinear (type of transformation specified) or categorized *A general statement is sufficient, no need to describe this for each predictor separately.  If no continuous predictors were reported, score Not applicable.* | Y | Not applicable | Y |
| ii | For categorical or categorized predictors, the cut-points were reported *If no categorical or categorized predictors were reported, score Not applicable.* | N | Not applicable | N |
| iii | For categorized predictors the method to choose the cut-points was clearly described *If no categorized predictors, score Not applicable.* | N | Not applicable | N |
| **10b** | **Specify type of model, all model-building procedures (including any predictor selection), and method for internal validation.** | 1 | NA | **1** |
| i | The type of statistical model is reported *E.g. Logistic, Cox, other regression model (e.g. Weibull, ordinal), other statistical modelling (e.g. neural network)* | Y | Not applicable | Y |
| ii | The approach used for predictor selection before modelling is described *‘Before modelling’ means before any univariable or multivariable analysis of predictor-outcome associations. If no predictor selection before modelling is done, score Not applicable. If it is unclear whether predictor selection before modelling is done, score No. If it is clear there was predictor selection before modelling but the method was not described, score No.* | Y | Not applicable | Y |
| iii | The approach used for predictor selection during modelling is described *E.g. Univariable analysis, stepwise selection, bootstrap, Lasso. ‘During modelling’ includes both univariable or multivariable analysis of predictor-outcome associations.  If no predictor selection during modelling is done (so-called full model approach), score Not applicable. If it is unclear whether predictor selection during modelling is done, score No.  If it is clear there was predictor selection during modelling but the method was not described, score No.* | Y | Not applicable | Y |
| iv | Testing of interaction terms is described *If it is explicitly mentioned that interaction terms were not addressed in the prediction model, score Yes.  If interaction terms were included in the prediction model, but the testing is not described, score No.* | Y | Not applicable | Y |
| v | Testing of the proportionality of hazards in survival models is described *If no proportional hazard model is used, score Not applicable.* | NA | Not applicable | NA |
| vi | Internal validation is reported  *E.g. Bootstrapping, cross validation, split sample. If the use of internal validation is clearly a non-issue (e.g. in case of very large data sets), score Yes. For all other situations an explicit mention is expected.* | Y | Not applicable | Y |
| **10c** | **For validation, describe how the predictions were calculated.** | NA | 1 | **1** |
| i. | It is described how predictions for individuals (in the validation set) were obtained from the model being validated  *E.g. Using the original reported model coefficients with or without the intercept, and/or using updated or refitted model coefficients, or using a nomogram, spreadsheet or web calculator.* | Not applicable | Y | Y |
| **10d** | **Specify all measures used to assess model performance and, if relevant, to compare multiple models.** *These should be described in methods section of the paper (item 16 addresses the reporting of the results for model performance).* | 1 | 1 | **1** |
| i | Measures for model discrimination are described *E.g. C-index / area under the ROC curve.* | Y | Y | Y |
| ii | Measures for model calibration are described *E.g. calibration plot, calibration slope or intercept, calibration table, Hosmer Lemeshow test, O/E ratio*. | Y | Y | Y |
| iii | Other performance measures are described  *E.g. R2, Brier score, predictive values, sensitivity, specificity, AUC difference, decision curve analysis, net reclassification improvement, integrated discrimination improvement, AIC.* | Y | Y | Y |
| **10e** | **Describe any model updating (e.g., recalibration) arising from the validation, if done.** | NA | NA | **NA** |
| i | A description of model-updating is given *E.g. Intercept recalibration, regression coefficient recalibration, refitting the whole model, adding a new predictor  If updating was done, it should be clear which updating method was applied to score Yes.  If it is not explicitly mentioned that updating was applied in the study, score this item as ‘Not applicable’.* | Not applicable | NA | NA |
| **11** | **Provide details on how risk groups were created, if done.** *If risk groups were not created, score this item as Yes.* | 1 | 1 | **1** |
| i | If risk groups were created, risk group boundaries (risk thresholds) are specified  *Score this item separately for development and validation if a study includes both development and validation. If risk groups were not created, score this item as not applicable.* | NA | NA | NA |
| **12** | **For validation, identify any differences from the development data in setting, eligibility criteria, outcome and predictors.** | NA | 1 | **1** |
| i | Differences or similarities in definitions with the development study are described *Mentioning of any differences in all four (setting, eligibility criteria, predictors and outcome) is required to score Yes.  If it is explicitly mentioned that there were no differences in setting, eligibility criteria, predictors and outcomes, score Yes.* | Not applicable | 1 | 1 |
| **Results** | |  |  |  |
| **13a** | **Describe the flow of participants through the study, including the number of participants with and without the outcome and, if applicable, a summary of the follow-up time. A diagram may be helpful.** | 1 | 1 | **1** |
| i | The flow of participants is reported | 1 | 1 | 1 |
| ii | The number of participants with and without the outcome are reported *If outcomes are continuous, score Not applicable.* | 1 | 1 | 1 |
| iii | A summary of follow-up time is presented *This notably applies to prognosis studies and diagnostic studies with follow-up as diagnostic outcome. If this is not applicable for an article (i.e. diagnostic study or no follow-up), then score Not applicable.* | NA | NA | NA |
| **13b** | **Describe the characteristics of the participants (basic demographics, clinical features, available predictors), including the number of participants with missing data for predictors and outcome.** | 1 | 1 | **1** |
| i | Basic demographics are reported | 1 | 1 | 1 |
| ii | Summary information is provided for all predictors included in the final developed/validated model | 1 | 1 | 1 |
| iii | The number of participants with missing data for predictors is reported | 1 | 1 | 1 |
| iv | The number of participants with missing data for the outcome is reported | 1 | 1 | 1 |
| **13c** | **For validation, show a comparison with the development data of the distribution of important variables (demographics, predictors and outcome).** | NA | 0 | **0** |
| i | Demographic characteristics (at least age and sex) of the validation study participants are reported along with those of the original development study | Not applicable | 1 | 1 |
| ii | Distributions of predictors in the model of the validation study participants are reported along with those of the original development study | Not applicable | 0 | 0 |
| iii | Outcomes of the validation study participants are reported along with those of the original development study | Not applicable | 1 | 1 |
| **14a** | **Specify the number of participants and outcome events in each analysis.** | 1 | NA | **1** |
| i | The number of participants in each analysis (e.g. in the analysis of each model if more than one model is developed) is specified | 1 | Not applicable | 1 |
| ii | The number of outcome events in each analysis is specified (e.g. in the analysis of each model if more than one model is developed) *If outcomes are continuous, score Not applicable.* | 1 | Not applicable | 1 |
| **14b** | **If done, report the unadjusted association between each candidate predictor and outcome.** | 1 | NA | **1** |
| i | The unadjusted associations between each predictor and outcome are reported *If any univariable analysis is mentioned in the methods but not in the results, score No.  If nothing on univariable analysis (in methods or results) is reported, score this item as Not applicable.* | Y | Not applicable | Y |
| **15a** | **Present the full prediction model to allow predictions for individuals (i.e., all regression coefficients, and model intercept or baseline survival at a given time point).** | 0 | NA | **0** |
| i | The regression coefficient (or a derivative such as hazard ratio, odds ratio, risk ratio) for each predictor in the model is reported | 1 | Not applicable | 1 |
| ii | The intercept or the cumulative baseline hazard (or baseline survival) for at least one time point is reported | 0 | Not applicable | 0 |
| **15b** | **Explain how to use the prediction model.** | 1 | NA | **1** |
| i | An explanation (e.g. a simplified scoring rule, chart, nomogram of the model, reference to online calculator, or worked example) is provided to explain how to use the model for individualised predictions. | 1 | Not applicable | 1 |
| **16** | **Report performance measures (with confidence intervals) for the prediction model.** *These should be described in results section of the paper (item 10 addresses the reporting of the methods for model performance).* | 1 | 1 | **1** |
| i | A discrimination measure is presented *E.g. C-index / area under the ROC curve.* | 1 | 1 | 1 |
| ii | The confidence interval (or standard error) of the discrimination measure is presented | 1 | 1 | 1 |
| iii | Measures for model calibration are described *E.g. calibration plot, calibration slope or intercept, calibration table, Hosmer Lemeshow test, O/E ratio.* | 1 | 1 | 1 |
| iv | Other model performance measures are presented *E.g. R2, Brier score, predictive values, sensitivity, specificity, AUC difference, decision curve analysis, net reclassification improvement, integrated discrimination improvement, AIC.* | 1 | 1 | 1 |
| **17** | **If done, report the results from any model updating (i.e., model specification, model performance, recalibration).** *If updating was not done, score this TRIPOD item as ‘Not applicable’.* | NA | NA | **NA** |
| 0 | Model updating was done *If "No", then answer 17i-17v with "Not applicable"* | Not applicable | NA | NA |
| i | The updated regression coefficients for each predictor in the model are reported  *If model updating was described as ‘not needed’, score Yes.* | Not applicable | NA | NA |
| ii | The updated intercept or cumulative baseline hazard or baseline survival (for at least one time point) is reported  *If model updating was described as ‘not needed’, score Yes.* | Not applicable | NA | NA |
| iii | The discrimination of the updated model is reported | Not applicable | NA | NA |
| iv | The confidence interval (or standard error) of the discrimination measure of the updated model is reported | Not applicable | NA | NA |
| v | The calibration of the updated model is reported | Not applicable | NA | NA |
| **Discussion** | |  |  |  |
| **18** | **Discuss any limitations of the study (such as nonrepresentative sample, few events per predictor, missing data).** | 1 | 1 | **1** |
| i | Limitations of the study are discussed *Stating any limitation is sufficient.* | 1 | 1 | 1 |
| **19a** | **For validation, discuss the results with reference to performance in the development data, and any other validation data.** | NA | 1 | **1** |
| i | Comparison of results to reported performance in development studies and/or other validation studies is given | Not applicable | 1 | 1 |
| **19b** | **Give an overall interpretation of the results considering objectives, limitations, results from similar studies and other relevant evidence.** | 1 | 1 | **1** |
| i | An overall interpretation of the results is given | 1 | 1 | 1 |
| **20** | **Discuss the potential clinical use of the model and implications for future research.** | 1 | 1 | **1** |
| i | The potential clinical use is discussed  *E.g. an explicit description of the context in which the prediction model is to be used (e.g. to identify high risk groups to help direct treatment, or to triage patients for referral to subsequent care).* | 1 | 1 | 1 |
| ii | Implications for future research are discussed *E.g. a description of what the next stage of investigation of the prediction model should be, such as ”We suggest further external validation”.* | 1 | 1 | 1 |
| **Other information** | |  |  |  |
| **21** | **Provide information about the availability of supplementary resources, such as study protocol, web calculator, and data sets.** | 1 | 1 | **1** |
| i | Information about supplementary resources is provided | 1 | 1 | 1 |
| **22** | **Give the source of funding and the role of the funders for the present study.** | **1** | **1** | **1** |
| i | The source of funding is reported or there is explicit mention that there was no external funding involved | 1 | 1 | 1 |
| ii | The role of funders is reported or there is explicit mention that there was no external funding | 1 | 1 | 1 |
|  |  |  |  |  |
|  |  |  |  |  |
|  | **Number of applicable TRIPOD items** | **30** | **28** | **34** |
|  | **Number of TRIPOD items adhered** | **23** | **20** | **25** |
|  | **OVERALL adherence to TRIPOD** | **0.77** | **0.71** | **0.74** |

*Supplementary Table 19 – Software packages*

| Task | Environment | Package/extension | Functions/modules |
| --- | --- | --- | --- |
| qMRI reconstruction | MATLAB v. R2017b, | hMRI (Tabelow et al., 2019) v. 0.2.0 for SPM12 v. 7487 | ‘Create hMRI maps’ |
| MRI resampling | Python v. 3.7.1 | Nibabel (Brett et al., 2019) v. 0.5.2 | ‘processing.resample_to_output’ |
| WM lesions detection | MATLAB v. R2017b | LST (Schmidt et al., 2012) v. 1.2.3 for SPM12 v. 7487 | ‘Determination of the optimal initial threshold’,  ‘Lesion segmentation (LGA)’ |
| HCs brain tissues segmentation | MATLAB v. R2017b | SPM12 v. 7487 | ‘Segment’ |
| MS patients brain tissues segmentation | MATLAB v. R2017b | USwithLesion (Phillips, Pernet, 2017) for SPM12 v. 7487 | ‘US with lesion’ |
| Bias field correction | Python v. 3.7.1 | SimpleITK (Lowekamp et al., 2013) v. 1.2.4 | ‘N4BiasFieldCorrectionImageFilter’ |
| MRI intensities normalisation | Python v. 3.7.1 | Numpy (Walt et al., 2011) 1.18.4 | ‘nanmean’, ‘nanstd’ |
| Radiomic features extraction | Python v. 3.7.1 | PyRadiomics (Van Griethuysen et al., 2017) 2.2.0 | ‘featureextractor .RadiomicsFeaturesExtractor’ |
| Preliminary features analysis | Python v. 3.7.1 | Scipy (Virtanen et al., 2020) v. 1.1.0 | ‘spearmanr’, ‘stats.mannwhitneyu’ |
|  |  | Statsmodels (Seabold et al., 2010) v. 0.9.0 | ‘multitest’ |
| Features selection | Python v. 3.7.1 | Scikit-learn (Pedregosa et al., 2011) v. 0.22.1 | ‘feature_selection.RFE’,  ‘ensemble .RandomForestClassifier’ |
| Models training | Python v. 3.7.1 | Scikit-learn v. 0.22.1 | ‘model_selection.StratifiedShuffleSplit’, ‘preprocessing.MinMaxScaler’,  ‘ensemble.RandomForestClassifier’, ‘svm.SVM’, ‘linear_model.LogisticRegression’, |
| Models testing/validation | Python v. 3.7.1 | Scikit-learn v. 0.22.1 | ‘accuracy_score’, ‘roc_auc_score, ‘recall_score’, |
| Operations with arrays | Python v. 3.7.1 | Numpy v. 1.18.4 |  |
| Operations with features dataframes | Python v. 3.7.1 | Pandas (McKinney et al., 2010) v. 1.0.3 |  |
| Plots | Python v. 3.7.1 | Seaborn (Waskom et al., 2017) v. 0.9.0  Matplotlib (Hunter et al., 2007) v. 3.1.3 |  |
| Read/write access to Nifti files | Python v. 3.7.1 | Nibabel 0.5.2 |  |

# References

Lommers, E., Simon, J., Reuter, G., Delrue, G., Dive, D., Degueldre, C., . . . Maquet, P. (2019). Multiparameter MRI quantification of microstructural tissue alterations in multiple sclerosis. Neuroimage Clin, 23, 101879. <https://doi.org/10.1016/j.nicl.2019.101879>

Van Griethuysen, J. J., Fedorov, A., Parmar, C., Hosny, A., Aucoin, N., Narayan, V., ... & Aerts, H. J. (2017). Computational radiomics system to decode the radiographic phenotype. Cancer research, 77(21), e104-e107. <https://doi.org/10.1158/0008-5472.CAN-17-0339>

Tabelow, K., Balteau, E., Ashburner, J., Callaghan, M. F., Draganski, B., Helms, G., ... & Reimer, E. (2019). hMRI – A toolbox for quantitative MRI in neuroscience and clinical research. Neuroimage, 194, 191-210. <https://doi.org/10.1016/j.neuroimage.2019.01.029>

Brett, Matthew, Markiewicz, Christopher J., Hanke, Michael, Côté, Marc-Alexandre, Cipollini, Ben, McCarthy, Paul, … freec84. (2019, April 2). nipy/nibabel: 2.4.0 (Version 2.4.0). Zenodo. <https://doi.org/10.5281/zenodo.2620614>

Schmidt, P., Gaser, C., Arsic, M., Buck, D., Förschler, A., Berthele, A., ... & Hemmer, B. (2012). An automated tool for detection of FLAIR-hyperintense white-matter lesions in multiple sclerosis. Neuroimage, 59(4), 3774-3783. <https://doi.org/10.1016/j.neuroimage.2011.11.032>

Phillips, C., & Pernet, C. (2017). Unifying lesion masking and tissue probability maps for improved segmentation and normalization. Available: <https://orbi.uliege.be/bitstream/2268/228841/1/OHBM17_Abstract_USwL_final_wFig.pdf>

Lowekamp, B. C., Chen, D. T., Ibáñez, L., & Blezek, D. (2013). The design of SimpleITK. Frontiers in neuroinformatics, 7, 45. <https://doi.org/10.3389/fninf.2013.00045>

Walt, S. V. D., Colbert, S. C., & Varoquaux, G. (2011). The NumPy array: a structure for efficient numerical computation. Computing in science & engineering, 13(2), 22-30. <https://arxiv.org/pdf/1102.1523.pdf%C3%AB%C2%A5%C2%BC>

Virtanen, P., Gommers, R., Oliphant, T. E., Haberland, M., Reddy, T., Cournapeau, D., ... & van der Walt, S. J. (2020). SciPy 1.0: fundamental algorithms for scientific computing in Python. Nature methods, 17(3), 261-272. <https://doi.org/10.1038/s41592-019-0686-2>

Seabold, S., & Perktold, J. (2010, June). Statsmodels: Econometric and statistical modeling with python. In Proceedings of the 9th Python in Science Conference (Vol. 57, p. 61). [https://doi.org/10.25080/10.25080/Majora-92bf1922-011](https://doi.org/10.25080/Majora-92bf1922-00a)

Pedregosa, F., Varoquaux, G., Gramfort, A., Michel, V., Thirion, B., Grisel, O., ... & Vanderplas, J. (2011). Scikit-learn: Machine learning in Python. Journal of machine learning research, 12(Oct), 2825-2830. Available: <https://arxiv.org/abs/1201.0490>

McKinney, W. (2010, June). Data structures for statistical computing in python. In Proceedings of the 9th Python in Science Conference (Vol. 445, pp. 51-56). <https://doi.org/10.25080/Majora-92bf1922-00a>

Waskom, M., Botvinnik, O., O’Kane, D., Hobson, P., Lukauskas, S., Gemperline, D. C., ... & de Ruiter, J. (2017). mwaskom/seaborn: v0. 8.1 (September 2017). Zenodo, doi, 10. <https://doi.org/10.5281/zenodo.883859>

Hunter, J. D. (2007). Matplotlib: A 2D graphics environment. Computing In Science Engineering 9, 3. 90-95. <https://doi.org/10.1109/MCSE.2007.55>
